# Biosynthesized Silver Nanoparticle (AgNP) From *Pandanus odorifer* Leaf Extract Exhibits Anti-metastasis and Anti-biofilm Potentials

**DOI:** 10.3389/fmicb.2019.00008

**Published:** 2019-02-12

**Authors:** Afzal Hussain, Mohamed F. Alajmi, Meraj A. Khan, Syed A. Pervez, Faheem Ahmed, Samira Amir, Fohad M. Husain, Mohd S. Khan, Gouse M. Shaik, Iftekhar Hassan, Rais A. Khan, Md. Tabish Rehman

**Affiliations:** ^1^Department of Pharmacognosy, College of Pharmacy, King Saud University, Riyadh, Saudi Arabia; ^2^Program in Translational Medicine, Peter Gilgan Centre for Research and Learning, The Hospital for Sick Children, Toronto, ON, Canada; ^3^Helmholtz Institute Ulm, Electrochemical Energy Storage, Ulm, Germany; ^4^Department of Physics, College of Science, King Faisal University, Al-Ahsa, Saudi Arabia; ^5^Department of Chemistry, College of Science & General Studies, Al Faisal University, Riyadh, Saudi Arabia; ^6^Department of Food Science and Nutrition, College of Food and Agriculture, King Saud University, Riyadh, Saudi Arabia; ^7^Protein Research Chair, Department of Biochemistry, College of Science, King Saud University, Riyadh, Saudi Arabia; ^8^Department of Zoology, College of Science, King Saud University, Riyadh, Saudi Arabia; ^9^Department of Chemistry, College of Science, King Saud University, Riyadh, Saudi Arabia

**Keywords:** silver nanoparticles (AgNPs), anti-metastasis, anti-biofilm, quorum sensing, molecular docking

## Abstract

Cancer and the associated secondary bacterial infections are leading cause of mortality, due to the paucity of effective drugs. Here, we have synthesized silver nanoparticles (AgNPs) from organic resource and confirmed their anti-cancer and anti-microbial potentials. Microwave irradiation method was employed to synthesize AgNPs using *Pandanus odorifer* leaf extract. Anti-cancer potential of AgNPs was evaluated by scratch assay on the monolayer of rat basophilic leukemia (RBL) cells, indicating that the synthesized AgNPs inhibit the migration of RBL cells. The synthesized AgNPs showed MIC value of 4–16 μg/mL against both Gram +ve and Gram -ve bacterial strains, exhibiting the anti-microbial potential. Biofilm inhibition was recorded at sub-MIC values against Gram +ve and Gram -ve bacterial strains. Violacein and alginate productions were reduced by 89.6 and 75.6%, respectively at 4 and 8 μg/mL of AgNPs, suggesting anti-quorum sensing activity. Exopolysaccharide production was decreased by 61–79 and 84% for Gram -ve and Gram +ve pathogens respectively. Flagellar driven swarming mobility was also reduced significantly. Furthermore, *In vivo* study confirmed their tolerability in mice, indicating their clinical perspective. Collective, we claim that the synthesized AgNPs have anti-metastasis as well as anti-microbial activities. Hence, this can be further tested for therapeutic options to treat cancer and secondary bacterial infections.

## Introduction

Cancer is one of the leading causes of global mortality due to its poor diagnosis in early stages, undefined specificity and severe side effects associated with existing chemotherapeutic drugs (Siegel et al., 2017). The immune system of cancer patients undergoing chemotherapy is often compromised. Such patients are prone to secondary infection by different pathogens including bacteria, viruses, and fungi. The concerning aspect related to secondary infection in cancer patient is of the emergence of antibiotic resistance (Khan and Rehman, 2016; Muteeb et al., 2017). Recently, a novel focus of research evolved for the design, synthesis and characterization of metal-based nanoparticles (NPs) to treat and target many disease conditions including cancer and secondary associated infections (Oves et al., 2018). Among noble metal nanomaterials, silver and gold nanoparticles attain a distinction due to their attractive physicochemical properties related to biological systems (Salata, 2004; Mody et al., 2010). Silver nanoparticles (AgNPs) are under extensive research for their contribution in different fields including healthcare, food packaging and environment (Calderón-Jiménez et al., 2017). The cytotoxic potential of silver nanoparticles (AgNPs) has been reported on different cancer cell lines including A549 (lung cancer) (Gengan et al., 2013), MCF-7 (breast cancer) (Jeyaraj et al., 2013b), HT29 (colon cancer) (Sanpui et al., 2011), and HeLa (cervical cancer) (Jeyaraj et al., 2013a). Moreover, the antimicrobial potential of AgNPs is well-documented in scientific literature as well as in traditional medicine (Choi et al., 2008; Rai et al., 2009; Zhang et al., 2010). The antimicrobial activity of AgNPs has been attributed to the release of biologically active silver ions upon ionization of silver in aqueous solution (Choi et al., 2008; Rai et al., 2009). Now researchers focus is to develop nanoparticles of displaying multiple functions, such as the selective killing of cancer cells, bactericidal and antibiofilm activity. The inclusion of these properties in a single nanoparticle will play potential role for the treatment of cancer and associated secondary infections.

Indeed, a robust, less complicated, and economical procedure under ambient environment, needed for the synthesis of silver nanoparticles. Assessing antibiotic resistance, biofilm, quorum sensing, molecular docking, anti-cancer properties are important for clinical management. Recently, the synthesis of nanostructured materials via microwave irradiation has been introduced (Ahluwalia and Kidwai, 2004). In comparison with conventional heating, microwave heating has unique effects such as rapid and homogeneous volumetric heating, high reaction rate, short reaction time, enhanced reaction selectivity, energy savings and low cost (Ahmed et al., 2011). It has already been reported that as compared to traditional heating, microwave heating creates nanoparticles of higher degree of crystallinity and narrower size distributions, besides granting greater control over the shape morphology of the nanostructures (Tsuji et al., 2005).

The synthesis of NPs from plant extract is an emerging novel approach to produce inexpensive and environment friendly nanoparticles with control size and crystallinity. Plants serve as an impeccable resources of biological samples and reducing agent for the synthesis of AgNPs (Jha et al., 2009), because of rich in alkaloids, saponins, tannins, vitamins, phenolics, and terpenoids organic components. Furthermore, these plant resources provide an inexpensive, efficient and eco-friendly, single-step procedure for the synthesis of AgNPs without involving any extrinsic surfactants, capping agents, and templates (Jha et al., 2009).

To achieve the goal of synthesizing eco-friendly, economicallay, having anti-cancer and anti-biofilm properties, under ambient conditons, we synthesized silver nanoparticles (AgNPs) by using *Pandanus odorifer* leaf extract (POLE) as a bio-template by microwave irradiation (MWI) method (Adkar and Bhaskar, 2014). The synthesized AgNPs were evaluated for their anti-cancer potential against rat basophilic leukemia (RBL) cell lines by MTT [3-(4,5-dimethylthiazol-2-yl)-2,5-diphenyltetrazolium bromide] and scratch assays. Moreover, the biosynthesized AgNPs were also proven as an antibacterial and antibiofilm agent against different Gram +ve and Gram -ve bacterial strains. Quorum sensing (QS) regulated phenomenon such as violacein, alginate and exopolysaccharide (EPS) productions, and biofilm formation were also evaluated in the presence of POLE-capped AgNPs. *In silico*, molecular modeling and docking studies were performed to evaluate the interaction of silver (Ag) with key proteins involved in quorum sensing. Additionally, *In vivo* animal toxicity and comet assay were performed to access the tolerability of AgNPs. This is first report on the synthesis of AgNPs using *P. odorifer* leaf extract by microwave irradiation and their assessment for biomedical applications. The synthesized nanoparticle and their characterized properties have many potential therapeutic option for the treatment of cancer and infectious disease states.

## Materials and Methods

### Ethics Statement

The study protocol and treatment methods (care and handling of experimental animals) were approved by the Animal Ethics Committee of the Zoology Department in the College of Science at King Saud University, Riyadh (Saudi Arabia).

### Materials and Reagents

Silver nitrate (AgNO_3_) was obtained from Sigma-Aldrich, United States. All other reagents and chemicals were purchased from Sigma, unless otherwise stated. Antibiotics were purchased from Invitrogen Life Technologies, United States. The plant (*P. odorifer*) leaf specimens were collected from the University campus in Aligarh, Uttar Pradesh, India for further use. Reagents were used without further purification unless described specifically.

### *Pandanus odorifer* Leaf Extract (POLE) Preparation

The leaf extract of the plant *P. odorifer* as prepared by fast pressurized solvent extraction using a Speed Extractor E-914 (Buchi, Germany). *P. odorifer* is a rich source of phytochemicals including lignans, isoflavones, coumestrol, alkaloids, steroids, phenolic compounds, glycosides, proteins, and various essential and non-essential amino acids (Adkar and Bhaskar, 2014). Fresh and healthy leaves of *P. odorifer* were washed thoroughly with plenty of distilled water, and both the surfaces of leaves were sterilized using 70% alcohol by gentle rubbing. The leaves were dried and crushed to powder. For the extraction and filtration of plant extract, speed extractor cell was filled with leaves powder (5 g) and sand (1 g) followed by the filter paper and metal frit. The speed extractor has been programmed for two cycles of 42 min at 50°C in 100 mL of water. After 42 min, the filtered plant extract (50 mg/mL concentration) was collected in the collection tray of the speed extractor and stored in cool and dry place for further use.

### Total Phenolic Content Analysis

Estimation of total phenolic content has been done using standard gallic acid curve as described earlier (Luximon-Ramma et al., 2002). Briefly, 0.125 ml of leaf extract (50 mg/mL) was mixed with 0.5 mL deionized water followed by the addition of 0.125 mL Folin-Ciocalteau reagent and incubated for 5 min at room temperature. 1.25 mL of Na_2_CO_3_ (7%) solution was added to the above mixture and made up to 3 mL with deionized water. After 90 min of incubation at room temperature, an absorption maximum was monitored at 760 nm.

### Total Flavonoid Analysis

The total flavonoid content was analyzed from a standard quercetin curve (Ghosh et al., 2012). 0.5 mL AlCl_3_ (2% in methanol) was mixed with 0.5 mL leaf extract sample (50 mg/mL) and incubated for 10 min at room temperature. After incubation at room temperature, the absorbance was recorded at 368 nm.

### Microwave Irradiation-Assisted Synthesis of POLE-Capped AgNPs

The stable AgNPs from *P. odorifer* leaf extract was prepared in a one-step microwave irradiation assisted synthesis guided by the green chemistry principles (Tsuji et al., 2005; Iravani and Zolfaghari, 2013; Mirgorod and Borodina, 2013; Mirgorod et al., 2013; Ali et al., 2015; Alajmi et al., 2018). The reaction conditions for microwave irradiation-assisted synthesis of POLE-capped AgNPs were optimized by varying the concentrations of AgNO_3_ (2–10 mM), POLE concentration (1–5 mL of 50 mg/mL), pH (5–8), and incubation time (0–30 min). Finally, 5 mL of AgNO_3_ solution (8 mM) was added dropwise to 5 mL of leaf extract (50 mg/mL) at pH 8.0 under stirring condition for 10 min. For microwave irradiation-assisted synthesis, the POLE-AgNO_3_ reaction mixture was kept in a domestic microwave [Sharp R-75AS(S)] operating under 900 W power and 2450 MHz frequency, for a pulse of 90 s and allowed for the cooling at room temperature. As a control, POLE-AgNPs were also synthesized conventionally by incubating the reaction mixture of POLE and AgNO3 for 4 h without microwave treatment.

### Biophysical Characterization of Synthesized Silver Nanoparticle

UV-Vis absorption spectrum of AgNPs was recorded in the range of 300–800 nm using a UV-Vis spectrophotometer (Evolution 201, Thermo Scientific). Distilled water was used as a reference or control. Futher the phase purity of AgNPs was determined by X-ray diffraction (XRD) using a Phillips-PW 1729 X-ray diffractometer (Holland). The material was investigated with Cu radiation (1.54430 Å). The XRD patterns were recorded with a step size of 0.02° and scan speed of 2°/min in the scan ranging from 30 to 80° of 2θ. The Fourier transform infrared spectroscopy (FTIR; Perkin-Elmer) spectra of the product were recorded in the range of 4000–400 cm^-1^. Raman spectra of the samples were obtained using Raman microscope (XploRA ONE from Horiba, Kyoto, Japan). Raman spectra were collected using 532 nm laser excitation (25 mW) at room temperature. High-resolution transmission electron microscopy (HRTEM) was performed using a JEOL JSM-2100F operating at 200 kV. For the HRTEM observations, a drop of the specimen dispersed in ethanol was placed on copper grids and dried.

### Culture of Rat Basophil Leukemia (RBL) Cells

To study the anti-cancer activity of synthesized nanoparticle, the rat basophil leukemia (RBL) cell model has been used (Siraganian et al., 1982; Draberova and Draber, 1991; Zuberi et al., 1994; Sun et al., 2015). RBL-2H3 cells were thawed by gentle agitation in a 37°C water bath and revived at 37°C and 5% CO_2_ in Eagle’s minimum essential medium (MEM) with Earl’s balanced salt solution and supplemented with 10% heat-inactivated fetal bovine serum (FBS). Cells were grown as monolayer and dissociated with 0.2% EDTA for further use and sub-culture.

### Cytotoxicity Assessment by MTT Assay

Cytotoxicity of AgNPs was assessed on RBL cells using MTT assay by measuring the metabolic activity of cells (Mosmann, 1983). RBL cells (20,000 cells/well) were seeded into the flat-bottom 96-well plates and cultured in DMEM supplemented with 10% FBS for 24 h at 37°C in 5% CO_2_ incubator. After 24 h of the culture, cells were treated with different dosages of AgNPs (2–12 μg/mL) in the growth medium for further 24 h under the same culture conditions. Wells having only cells (media as a vehicle only) without nanoparticles were treated as a negative control, while the wells with only MTT, considered as a positive control. Cell viability was determined by adding MTT dye (100 μl of 0.1 mg/mL stock) to respective well and incubated for 4 h at 37°C with 5% CO_2_ in the dark. 3-(4,5-dimethylthiazol-2-yl)-2,5-diphenyltetrazolium bromide (MTT) a yellow tetrazole reduced to purple formazan in living cells by the activity of NAD(P)H-dependent cellular oxidoreductase enzymes. The insoluble formazan crystals were dissolved in isopropanol for 1 h at 37°C, into a colored solution. The quantification of formazan was performed by measuring absorbance at 570 nm. The degree of light absorption depends on the solvent. The percentage cell viability was calculated by taking (absorbance of the sample)/(absorbance of control) × 100. Cell toxicity is defined as 100-percentage cell viability.

### *In vitro* Scratch Assay

To see the effect of AgNPs on the cell–cell interaction, and cell migration aspects of cancer/metastasis, we performed the *in vitro* scratch assay by culturing RBL cells (Rodriguez et al., 2005). Equal numbers of RBL cells were seeded into respective wells of 24 well culture plates and grown to monolayer confluency. A scratch was made in the monolayer with the help of sterile pipette tip. Precaution was taken to maintain the same angle for test and control wells. After making a scratch, cells were washed briefly to decant unattached cells. At 10X magnification of Leica microscope equipped with a camera, images of control (Culture medium) and test wells (culture medium supplemented with 3 μg/mL AgNPs) were captured at 0 h and at a regular interval of 24 h, up to 72 h. Further the captured images were analyzed by ImageJ software (NIH, open source).

### Minimum Inhibitory Concentration (MIC) Assay

Minimum inhibitory concentration (MIC) is defined as the minimum concentration of chemical/drug agent, which prevent visible growth of the test strain of the bacteria. Here, we have determined MICs of AgNPs against the bacterial pathogens using micro-broth dilution method, following Clinical Laboratory Standards Institute (SLI) (Yamaguchi et al., 2012; Husain and Ahmad, 2013). Concentrations below the MICs were considered sub-inhibitory and were further used to study the anti-QS and biofilm inhibitory properties of the synthesized AgNPs. All experiments were performed in the BSL-2 safety class laboratory.

### Violacein Inhibition Assay

*Chromobacterium violaceum-*produces violacein, a water-insoluble purple pigment, that used to assess the activity of compound (Al-Shabib et al., 2016; Kothari et al., 2017). We have tested the violacein production by *C. violaceum* (CV12472) in the presence of AgNPs. The *C. violaceum* culture supplemented with C6-HSL (10 μM) in the presence and absence of AgNPs was grown overnight. Overnight grown cells were harvested and centrifuged at 1300 × *g* for 10 min, and the pellet was dissolved in 1 mL DMSO. The solution was vortexed vigorously for 30 s to completely solubilize violacein and again centrifuged. The absorbance of the soluble violacein was read at 585 nm using microplate reader (Thermo Scientific, Multiskan Ex, India). The percentage reduction of violacein production in the presence of AgNPs was calculated as (OD of control – OD of treated)/(OD of control) × 100.

### Extraction and Quantification of Exopolysaccharides (EPS)

Many Gram -ve and Gram +ve bacteria secret EPSs (high molecular weight polymers), a component of biofilm into their surrounding environments (Kothari et al., 2017). To test the anti-biofilm activity of the synthesized nanoparticles, EPSs estimation has been done in presence and absence of AgNPs during biofilm formation by different bacteria. Different bacterial strains were grown on nutrient agar plates in presence and absence of sub-MICs of synthesized AgNPs. The grown samples were picked, centrifuged, and the resulting supernatant was filtered. Three volumes of chilled 100% ethanol were added to the filtered supernatant and incubated overnight at 4°C to precipitate the EPS. EPS was then quantified by measuring sugars following the phenol method (Dubois et al., 1956; Vijayabaskar et al., 2011).

### Alginate Inhibition Assay

An overnight culture (1% v/v) of *Pseudomonas aeruginosa* was added to Luria-Bertani broth medium supplemented with or without AgNPs (1–8 μg/mL) and further cultured for overnight at 37°C in shaking condition (Gopu et al., 2015). To estimate the alginate production, 600 μl of boric acid-sulfuric acid solution (4:1) was added into 70 μl of AgNP either treated or untreated with bacteria in ice bath. The mixture was placed back again in an ice bath after vortexing for 10 s. Further, 20 μl of 0.2% carbazole dissolved in ethanol was added to the above mixture and vortexed for 10 s. The mixture was incubated for 30 min at 55°C and quantified at 530 nm using a microplate reader.

### Swarming Motility Assay

Swarming motility was determined as reported previously (O’Toole and Kolter, 1998). Briefly, an overnight culture of test pathogens was point inoculated at the center of the medium consisting of 1% tryptone, 0.5% NaCl and 0.3% agar with or without sub-MICs of synthesized AgNPs.

### Assay for Biofilm Inhibition

The effect of AgNPs on biofilm formation was measured using the polyvinyl chloride biofilm formation assay (O’Toole and Kolter, 1998). The overnight cultures of different pathogens were re-suspended in fresh LB medium in the presence and the absence of AgNPs and incubated at 30°C for 24 h. The biofilms in the microtiter plates were stained with a crystal violet solution and quantified by solubilizing the dye in ethanol and absorbance recorded at 470 nm.

### Assessment of *in vivo* Toxicity

All the animal-based experiments were conducted in accordance with the guidelines for the care and use of experimental animals by the *Committee for the Purpose of Control and Supervision of Experiments on Animals* and the *National Institutes of Health*.

Twenty-four adult Swiss albino rats (100–120 g, 3–4 months old) were purchased from the central animal house, Department of Pharmacy, King Saud University, Riyadh, Saudi Arabia. All the animals were randomly divided into four groups (*n* = 6) -ve control (vehicle treated) denoted as CN-, while CCl_4_ treated rats were considered as +ve control and denoted as CN+ (Shruti et al., 2015). CCl4 was administered as a single dose (1 mL/kg) dissolved in liquid paraffin in the ratio of 1:1 by volume. The AgNPs were injected intraperitoneally four times (once a week) at the dosage of 1 and 2 mg/kg of body weight and grouped as NP-1 and NP-2 respectively. All the rats were sacrificed on the same day by cervical dislocation after completion of the treatment. Their fresh liver and kidney tissues were subjected to sampling for comet assay and biochemical analyses. Blood was collected without anticoagulant in the BD Vacutainer^®^Blood Collection Tubes – BD and stored in the cold for serum analyses.

### Estimation of Liver Function Markers

Liver samples were homogenized and spun at 3000 × *g* in Tris-HCl buffer (pH: 7.4, 0.1 M), after spin the supernatants were collected for biochemical analyses. The serum was collected from the blood samples. The activity of liver marker enzymes such as aspartate aminotransferase (AST) and alanine aminotransferase (ALT) was estimated in the serum samples by commercially available kits (QCA, Spain). The estimation of the enzymes were performed by following the manufacturer’s protocol and otherwise mentioned.

### Evaluation of Kidney Function Markers

Kidney tissue samples were homogenized separately, spun at 3000 × *g* in Tris-HCl buffer (pH: 7.4, 0.1 M) and their supernatants were collected for biochemical analyses. The level of kidney function markers (urea and creatinine) was measured in the serum samples with the help of commercial kits (Linear, Spain) following the provided kit’s procedural instructions.

### Comet Assay

The target organs (liver and kidney) from each group were minced in RPMI 1640 medium and PBS (1:1 ratio) in a separate Petri dish (Corning, NY, United States). The very fine minced tissue released cells turned into a cell suspension, sieved through a muslin cloth and stored in Eppendorf vials (Eppendorf, Germany) at 4°C. The viability of the cells was checked by trypan blue exclusion method.

The protocol of Singh et al. (1988) with few modifications (Hassan et al., 2012) was used to perform comet assay in alkaline condition. Fully frosted slides pre-coated with 1% normal melting agarose (as a base layer) at 60°C was prepared a day before sacrificing the animals. About 10,000 cells isolated from each organ cell suspension was mixed with 100 mL of low melting agarose (1%) to form the working cell suspension separately for each organ. This suspension was pipetted over the base layer at 37°C followed by covering with coverslips immediately. After solidification of the second layer, they were kept on ice packs; the coverslips were removed followed by pouring a third layer of 0.5% low melting agarose (90 μL) and covering with coverslips on ice packs again. Followed by removal of the coverslips, the slides were dipped in a cold lysing solution at pH 10 for 3 h. Then, they were allowed to unwind in alkaline electrophoretic running buffer (300 mM NaOH + 1 mM EDTA) having pH 13 in the electrophoretic tank (Major Scientific, United Kingdom) for 30 min. Then, electrophoresis of the slides was performed for 35 min in cold condition at a constant field strength of 0.74 volts/cm varying the current strength of 300–310 mA. After gently washing with cold saline thrice, the slides were subjected to neutralizing buffer (0.4 M Tris-base) of pH 7.4 followed by washing with cold saline. The process of neutralization after washing was repeated thrice. 90 μl of ethidium bromide (stock of 20 mg/mL) was poured on each slide for staining of the nuclear DNA for 5 min. Followed by the slides were washed with chilled saline for three times and covered with coverslips. The slides were kept in a humidified slide box in the refrigerator and were analyzed on the next day by a fluorescent microscope (Leica, Germany) with 510–560 and 590 nm barrier filters. The microscope was coupled with an image analysis system (Komet 5.5, Kinetic imaging, Liverpool, United Kingdom) attached to integrated CC camera Zyla 5.5 (Andor, United Kingdom). The comets were scored at the magnification of 100X taking images of 50 cells for each treatment group (*n* = 2). In the present analysis, the Olive tail movement (migration of DNA from its nucleus in μm) was chosen to assess the nuclear DNA damage.

### Sequence Retrieval, Homology Modeling, and Molecular Docking

In *Pseudomonas aeruginosa*, LasR, Vfr, QscR, RhlR, and PqsA play a significant role in the formation of biofilm and QS. The FASTA sequences of these proteins were retrieved from NCBI database^1^. The Swiss Prot accession numbers of these proteins were P25084, P55222, G3XD77, P54292, and Q9I4X3 for LasR, Vfr, QscR, RhlR, and PqsA, respectively. A search in PDB database^2^ shows that the crystal structure of only LasR (4NG2), Vfr (2OZ6), and QscR (3SZT) were available. The sequences of the proteins with no crystal structures (RhlR and PqsA) were subjected to homology modeling using I-TASSER server (Roy et al., 2010). I-TASSER produced the three-dimensional models of RhlR and PqsA after threading based alignment and repeated structural compiling simulation. The quality of the predicted structures was validated by Ramachandran plot using RAMPAGE tool (Lovell et al., 2003). Further, these models were evaluated at QMEAN and SAVES servers. SAVES server runs six different tools namely CRYST1, PROVE, ERRAT, VERIFY3D, PROCHECK, and WHATCHECK to access protein structure (Pontius et al., 1996). These three-dimensional structures of these proteins were taken as the target for the purpose of molecular docking with Ag using PatchDock server (Schneidman-Duhovny et al., 2005). The sdf file of silver is downloaded from PubChem database^3^ and converted to pdb file using Discovery Studio 4.0 (Accelrys Software Inc., 2013). The PDB files of the respective protein and silver were uploaded under receptor–ligand interaction mode with default values, and RMSD cutoff value of <4.0 Å. Based on the scoring, the top-ranked solution was selected for interpretation. The amino acid residues of the proteins that are within 4 Å were analyzed for the molecular interaction with silver.

### Statistical Analysis

The data obtained from each experiments were presented as mean ± standard error values. The difference between control and test were analyzed using the Student’s *t*-test or otherwise stated in their respective legends. For animal studies, the results are expressed as a mean ± standard error of the mean (SEM) for six different samples taken in duplicate. Their statistical significance was evaluated by one-way ANOVA with Bonferroni post-test. For statistical comparison among the data of treatment groups, Tuckey’s test was followed by GraphPad Prism5 software under *in vivo* studies. The significance (*P*-values) and technical/biological repeats (*n*-values) were stated in repective figure legend.

## Results and Discussion

### Leaf Extract of *P. odorifer* Contains Flavonoids and Phenolic Components

The use of plant extract over microorganism for the synthesis of NPs is advantageous due to less biosafety issues in handling and disposal as compared to microorganisms. Moreover, keeping in mind that plant leaves are a rich source of flavonoids and phenolic components which can trigger the reduction of AgNO_3_ and control the size of synthesized AgNPs. We have used the leaf extract as one of the important ingredient for the synthesis of nanoparticles. First of all the aqueous extract of *P. odorifer* leaves was prepared and their flavonoids and phenolic components has been quantified. The OD based quantification data showed the presence of phenolic (0.105% wt/wt) and flavonoids (0.036% wt/wt) in the aqueous leaf extract of *P. odorifer*. Because of identifying the flavonoids and phenolic components in th eleaf extract, we further used this plant maerial for the synthesis of AgNPs. It is believed that free hydroxyl and carboxylic groups of flavonoids or phenols present in the plant extract may bind to the surface of Ag+ and trigger the formation of AgNPs while C O, C O–C, and C C groups of heterocyclic compounds may act as a stabilizer (Atwan and Saiwan, 2010; Awwad et al., 2012; Mukherjee et al., 2012).

### Ratio of Leaves Extract to Silver Nitrate Important for AgNPs Synthesis

**FIGURE 1:**
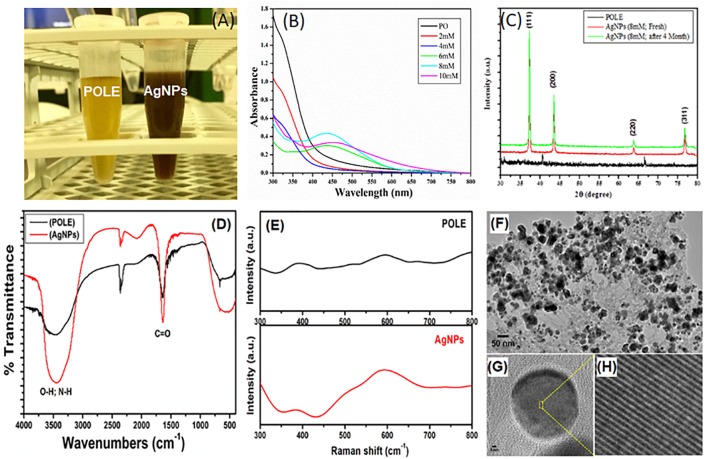
Characterization of POLE-capped silver nanoparticles (AgNPs). **(A)** Different concentrations of AgNO_3_ (2, 4, 6, 8, and 10 mM) were used to synthesize the AgNPs. 5 mL of each leaf extract (50 mg/mL) and AgNO_3_ solution (8 mM), gave best yield of silver nanoparticles by using microwave irradiation method. The resulting solution turned out to the characteristic brown color of the AgNPs in 90 min. **(B)** The absorption spectra of the synthesized nanoparticles were recorded in 300–800 nm wavelength range. The characteristic absorption peak of AgNPs is centered on 430 ± 20 nm. **(C)** The XRD data show diffraction peaks at 2θ = 37.2, 43.4°, 63.5, and 76.6° corresponding to the [111], [200], [220], and [311] planes of silver, respectively. **(D)** FT-IR spectra of plant extract (POLE) and AgNPs. The characteristic peak at 1636 cm^-1^ can be attributed to the C = O (carbonyl) groups. **(E)** Surface enhanced Raman spectroscopy (SERS), spectra displayed the characteristic signals of POLE with the enhanced intensity for AgNPs indicating the capping of POLE on the surface of AgNPs. **(F)** TEM image of POLE-capped AgNPs, **(G,H)** HRTEM (high-resolution transmission electron microscopy) images of POLE-capped AgNPs, clearly demonstrated the stability of the particles even after a period of 4 months.

It is always important to get a better yield and quality of the synthesized nanopartiles, which can be used for the clinical and commercial purposes. The concentration of plant extract having different amount of phenolics and flavonoids, plays an essential role in the synthesis of stable AgNPs. We for the first time optimized the ratio of *P. odorifer* leaves extract to silver nitrate for the synthesis of AgNPs nanoparticles. We found that the 5 mL of the leaf extract (50 mg/mL; total 250 mg) to 5 mL of AgNO_3_ solution (8 mM), gave best yield of silver nanoparticles by using microwave irradiation method. At this ratio of plant leaves extract and AgNO_3_, the resulting solution turned out to brown color, which is a characteristic of AgNPs (Figure 1A). AgNPs were also synthesized by using a range of the amount of leaf extract (1–4 ml), however, the resulting yield of AgNPs obtained was much lower. This might be due to insufficient flavonoids and phenolic components present in 1–4 mL of leaf extract, to completely reduce 5 mL of AgNO_3_ (8 mM) into AgNPs. Further, the flavonoids and phenolic components present in 5 mL of leave extract have enough threshold to reduce all Ag^+^ ions in the reaction mixture. Earlier a study performed by Panda et al. (2011) have used much less plant material (5% at v/v ratio 1:20) to reduce 1 mM AgNO_3_ than the amount we identified in this study (5% at v/v ratio 1:1) (Panda et al., 2011). Though the same study has used spathe (bract) from the male inflorescence of *P. odorifer* plant, which is altogether different source of the plant. This plant source may contain higher concentrations of flavonoids and phenolic components. However, the method adopted for the synthesis of nanoparticles by Panda et al. (2011) was time-consuming, has limitation in the availability of plant resource and the stability of the synthesized particles not known. In the present study, we have reported the synthesis of AgNPs via microwave irradiation assisted route which is cost-effective, energy saving, and faster process. These AgNPs were formed only within 90 s of microwave irradiation and are stable up to more than 4 months (Supplementary Figure S1).

### Synthesized AgNPs Showed Characteristic Absorption Peak in UV-Vis Absorption Spectra

After synthesizing the AgNPs, by using the leave extract as an economical and easily accessible green ingredient, we characterized the synthesized nanoparticles for their further use in clinical or commercial purposes. UV-Vis spectroscopy is a widely used technique to characterize the optical properties of the synthesized AgNPs. Different concentrations of AgNO_3_ (2, 4, 6, 8, and 10 mM) were used to synthesize the AgNPs and studied for the UV-Vis absorption spectra. The absorption spectra were recorded in 300–800 nm wavelength range. Figure 1B shows the UV-Vis absorption spectra of AgNPs prepared from different concentrations (of AgNO_3_ and 5 mL of POLE under MWI for 90 s). The appearance of an absorption band is due to the presence of free electrons in AgNPs, which mutually vibrate in resonance with the incident light wave thus yield an SPR (Surface Plasmon Resonance) absorption band. The characteristic absorption peak of AgNPs is centered on 430 ± 20 nm, depending on the size and polydispersity of the NPs (Anandalakshmi et al., 2016). In this study, the appearances of a single peak at approximately 435 nm indicated the formation of uniform AgNPs of 10–50 nm size. Interestingly, it has been observed that there was no AgNPs formation of up to 4 mM of silver nitrate concentration; however, once the concentration of silver nitrate has been increased to 6–8 mM, a prominent peak of AgNPs was observed. Moreover, on further increasing the concentration of silver nitrate up to 10 mM, a peak of AgNPs has been observed but with reduced intensity. This reduction of maxima absorption might be due to the aggregation of AgNPs owing to the limited concentration of plant extract. These results indicate that 5 mL of POLE is sufficient to reduce up to 8 mM Ag^+^ ions. Additionally, UV-Vis spectrum of the AgNPs was recorded at different time intervals for later time points up to 4 months. It entails that AgNPs were stable for up to 4 months at 8 mM concentration without losing the yield and absorption maxima (Supplementary Figure S1). Hence, these findings indicate that POLE provided stability to the AgNPs could be by acting as a binding or capping agent.

### X-Ray Diffraction (XRD) Determine the Crystalline Structure of ANPs

The crystalline phase of the synthesized AgNPs was determined by evaluating the X-ray diffraction (XRD) pattern. Figure 1C shows a typical XRD patterns of AgNPs, template hybrids (dried precursor, POLE) at room temperature and sintered AgNPs product after 4 months to demonstrate the stability of AgNPs. The XRD data show diffraction peaks at 2θ = 37.2, 43.4, 63.5, and 76.6° corresponding to the [111], [200], [220], and [311] planes of silver, respectively. Collectively the XRD data and pattern confirmed the crystalline structure of AgNPs. No peaks corresponding to other impurity crystalline phases were detected, and all the peaks in the XRD pattern can be readily indexed to the face-centered cubic (FCC) structure of silver (Ag). From the patterns, there was no change in 2θ values as the time passes, which evidenced about the stability of synthesized nanostructures.

### FTIR Shows the Capping of POLE on the Surface of AgNPs

To study the capping ability of plant extract on the surface of AgNPs, FTIR (Fourier-transform infra-red spectroscopy) studies were carried out. The FTIR spectrum of biosynthesized AgNPs by the POLE extract has been shown in Figure 1D. The spectrum showed a significantly broad and intense band at 3447 cm^-1^ associated with the stretching vibration of -OH (hydroxyl) and -NH (amine) groups of the POLE. The characteristic peak at 1636 cm^-1^ can be attributed to the C = O (carbonyl) groups. Thus, FTIR spectrum revealed that -C = O, -OH, and -NH functional groups were responsible for the reduction of Ag^+^ to Ag° and the broadening in the peaks of AgNPs spectrum confirmed the capping of POLE on the surface of AgNPs. This capping of POLE onto the AgNPs may be helpful in stabilizing nanoparticles and preventing agglomeration and leaching in the medium (Awwad et al., 2012; Chung et al., 2016). Furthermore, complementary studies on the capping of plant extract on the AgNPs surface were also carried out using SERS (surface enhanced Raman spectroscopy) studies. Figure 1E shows the SERS spectra of pristine plant extract (POLE) and AgNPs with POLE. SERS spectra displayed the characteristic signals of POLE with the enhanced intensity for AgNPs indicating the capping of POLE on the surface of AgNPs (Guo et al., 2016).

### Morphology and Stability of AgNPs

The detailed morphology of AgNPs were investigated by analyzing HRTEM (high-resolution transmission electron microscopy) images. HRTEM images of AgNPs synthesized freshly using 8 mM AgNO_3_ with MWI for 90 s and after 4 months storage was taken and presented in Figure 1F–H and Supplementary Figure S2, respectively. As shown in Figure 1F–H, AgNPs of 5 to 9 nm in size were uniformly dispersed. Moreover, the HRTEM images in Supplementary Figure S2 clearly demonstrated the stability of the particles even after a period of 4 months. Although, the stability of AgNPs remains a vital factor in determining the anti-microbial activity of AgNPs, there are only a few attempts in improving their stability. It has been recently reported that the stability of AgNPs and hence their biological activity can be improved manifolds by using plant extract as reducing agent and stabilizer (Qu et al., 2014). All these results indicate the successful preparation and stability of AgNPs.

### MTT and Scratch Assay Establish the Anti-cancer Activity of AgNPs

**FIGURE 2:**
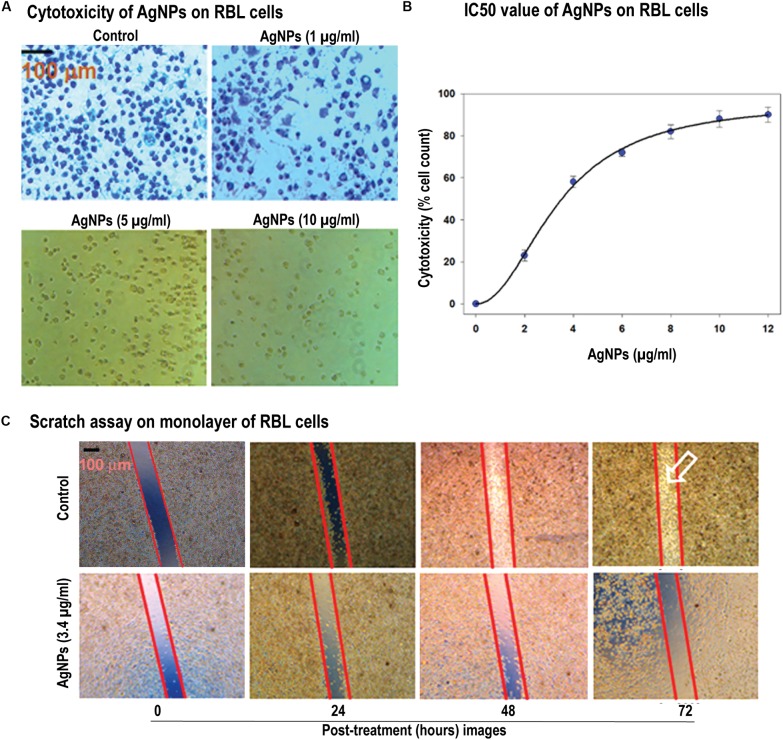
Cellular toxicity and cell migration in response of AgNPs. **(A)** Cytotoxicity of AgNPs on RBL cells were determined by MTT assay. MTT salt gets reduced by reducing enzymes into water-insoluble formazan. The images of the cells with AgNPs showed the dosage dependent effect of the AgNPs (1–10 μg/mL). **(B)** Determination of IC_50_ value (estimated to be 3.40 μg/mL) of AgNPs on RBL cells (data represented as SEM). **(C)** Scratch was made onto a monolayer of RBL cells and treated by 3 μg/mL of AgNPs (denoted as T, while the control as C) for different time points. Comparison of migration in both C and T was made by taking images at different time intervals (0, 24, 48, and 72 h) [control -(i), (ii), (iii), (iv) and test -(v), (vi), (vii), (viii)]. Results represented by marking the scratch with parallel lines and visually displaying the number of cells migrated in to the scratch area. The scale bar in **(A,C)** represents 100 μm.

After confirming the quality of the synthesized nanoparticles, we were asked to check the biological features of the AgNPs. Therefore, we assessed the cytotoxicity of AgNPs, using a colorimetric method based on MTT [3-(4,5-dimethylthiazol-2-yl)-2,5-diphenyl tetrazolium bromide]. The assay indirectly measures the mitochondrial activity of viable cells as a function of cell growth and proliferation. MTT salt gets reduced by reducing enzymes such as mitochondrial dehydrogenases of biologically active cells into water-insoluble formazan. In this study, MTT assay was performed to evaluate the cytotoxic effect of AgNPs on RBL cells (Figure 2A,B). The results indicate that AgNPs were cytotoxic toward these cells in a concentration dependent manner with an IC_50_ value of 3.40 μg/mL. The cytotoxicity of AgNPs is well-established and depends on nature of cell types and size of nanopartilces (Park et al., 2011). Cytotoxicity of AgNPs has been reported on many cancer cell lines including MDA-MB-231 breast cancer cells (Franco-Molina et al., 2010), and human Chang liver cells (Piao et al., 2011). It is interesting to note that similar IC_50_ values have been reported for AgNPs on human-derived keratinocyte HaCaT cell line (IC_50_ 6.8 ± 1 μM) (Zanette et al., 2011) and MDA-MB-231 cells (IC_50_ 8.7 μg/mL) (Gurunathan et al., 2013). Furthermore, it is reported that AgNPs are selectively more toxic toward cancerous cells than normal cells (Gopinath et al., 2008; Arora et al., 2009; Faedmaleki et al., 2014, 2016). Gopinath et al. (2008) investigated the molecular mechanism of AgNP (10–15 nm size) mediated cytotoxicity in BHK21 (non-cancer) and HT29 (cancer) cells, and they observed that AgNPs were selectively cytotoxic toward cancer cells. Faedmaleki and co-workers have reported that the IC_50_ of AgNPs on liver primary cells of mice was 121.7 μg/mL (Faedmaleki et al., 2014), while the same NPs were highly cytotoxic toward HepG2 cells (IC_50_ 2.8 μg/mL) (Faedmaleki et al., 2016). While Arora et al. (2009) has found that AgNPs were not-cytotoxic toward primary mouse fibroblasts (IC_50_ 61 μg/mL) and primary liver cells (IC_50_ 449 μg/mL). Keeping this in mind, we evaluated the cytotoxic effect of POLE-capped AgNPs on RBL cell line only and found that these NPs were highly cytotoxic (IC_50_ 3.40 μg/mL). The lower IC_50_ value in this study might be due to smaller size and different surface properties of AgNPs.

Cell migration plays an indispensable role in the progression of cancer. In this study, we have used *in vitro* scratch assay on RBL cells as it’s a robust and most commonly used method to probe cell migration (Liang et al., 2007). We have observed that AgNPs at 3 μg/mL concentration were effective in inhibiting cell migration in scratch plate assay (Figure 2C). As cytoskeleton rearrangements are crucial for cell migration, it is worth to speculate that AgNPs might interfere with this process supporting previously published data (Xu et al., 2013; Cooper and Spitzer, 2015). Also, cell division requires cytoskeleton rearrangement, and AgNPs effect on cell proliferation can also be seen in Figure 2Cvii,viii images, where a number of cells compared to control are diminished. The synthesized AgNPs show the notable effect on cell proliferation and migration. Owing to its effect at low concentrations this could be a candidate for further experiments *in vivo* on cancer models. Our results agree with previous literature where they have reported nanoparticles inhibit the migration of cancer cells (Shruti et al., 2015; Buranasukhon et al., 2017).

### AgNPs Show the Anti-microbial Activity

Very first we determined the MIC of AgNPs against pathogenic bacteria by micro-broth dilution method, and the results were interpreted according to CLSI, 2017 (Clinical and Laboratory Standards Institute) (CLSI, 2017) guidelines with some modifications (Andrews, 2001). Lowest concentration showed complete inhibition of visible microbial growth was considered as the MIC. In the present study, the MICs of AgNPs against *C*. *violaceum* CV12472, *P. aeruginosa* PAO1, *K. pneumoniae* ATCC 700603, *E. coli* ATCC 25922, and *S. aureus* ATCC 25923 strains were determined, and the results are presented in Table 1. The effect of AgNPs on the growth kinetics of test pathogens was determined using both growth curves and colony forming unit (CFU) values at 1/2 × MIC values. Selected sub-inhibitory concentrations did not show significant effect on the growth of the pathogens as shown in Supplementary Figures S3, S4.

**Table 1:** Minimum inhibitory concentrations (MICs) of AgNPs against different pathogens.

Strains	MIC of AgNPs (μg/mL)	Sub-MICs of AgNPs selected for assays (μg/mL)			
		
	MRSA	1/16 × MIC	1/8 × MIC	1/4 × MIC	1/2 × MIC
*P. aeruginosa*	16	1	2	4	8
*E. coli*	4	0.25	0.5	1	2
*C. violaceum*	8	0.5	1	2	4
*K. pneumoniae*	16	1	2	4	8
*S. aureus*	4	0.25	0.5	1	2


### AgNPs Exhibit Anti-quorum Sensing (QS) Activity

**FIGURE 3:**
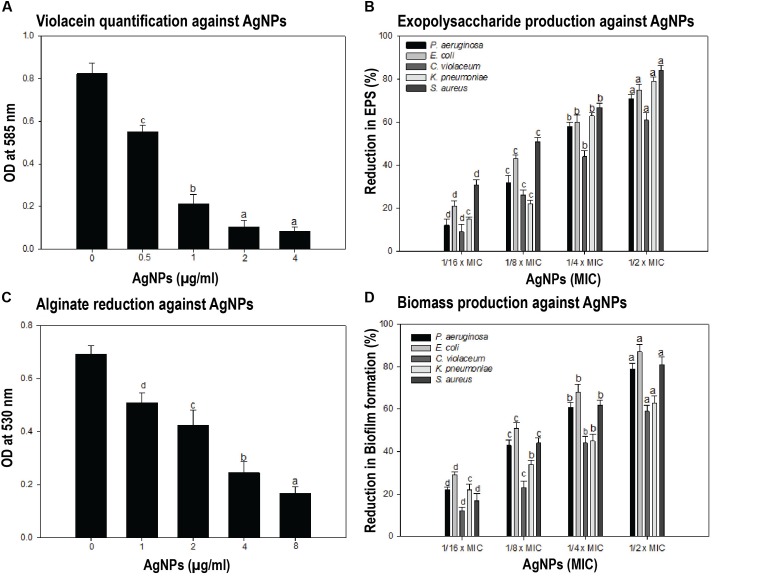
Anti-quorum sensing (QS) and anti-biofilm properties of AgNPs. **(A)** To test the effect of synthesized nanoparticles during the process of quorum sensing, the violacein was quantified by taking the OD at 585 nm in different conditions. Different concentrations of AgNPs showed the significant dosage dependent manner reduction in violacein production by *Chromobacterium violaceum*. Data showed the QS-mediated inhibition of violacein by AgNPs. **(B)** The inhibition of biofilm formation assessed by estimating the exopolysaccharide (EPS) production in presence and or absence of different concentrations of AgNPs was performed. Data showed the reduction in EPS production with increasing AgNPs concentration. **(C)** The reduced alginate production in response to different concentrations of AgNPs has been quantified. The presence of synthesized AgNPs showed 26.3–75.6% reduction in alginate production by *Pseudomonas aeruginosa.* **(D)** The biofilm formation was assessed directly by taking OD at 470 nm in microtiter plate. AgNPs presence causes the reduction of biofilm biomass production in the range of 22–79, 29–87, 12–59, 22–63, and 17–81% by *P*. *aeruginosa*, *E. coli*, *C. violaceum*, *K*. *pneumoniae*, and *S. aureus* respectively. One-way ANOVA with Dunnet post-test for **(A,C)**, in comparison to zero dosage of AgNPs, while one-way ANOVA with multiple comparison applied in **(B,D)**. Means denoted by the same letter within parameter are not significantly different at *p* ≤ 0.05, using DMRT.

Quorum sensing (QS) is a bacterial cell–cell communication process, in which bacteria response to extracellular signaling molecules to control microbial virulence. We were interested to determine the effect of synthesized AgNPs in relation to quorum sensing activity and biofilm formation. The anti-QS potential of AgNPs was accessed on *C. violaceum* CV12472 strain by observing the inhibition of QS-induced violacein (violet color pigment) formation. Quantification of violacein was performed spectrophotometrically using microtiter plate at 585 nm optical density (Figure 3A). Different concentrations of AgNPs exhibited a statistically significant reduction in the purple colored violacein production without inhibiting bacterial growth. At the lowest concentration (0.5 μg/mL), nanoparticles inhibited violacein production up to 32.9% as compared to control (*p* < 0.05). Violacein production decreased gradually with increasing concentration of AgNPs to a maximum of 89.6% at the concentration of 4 μg/mL (*P* < 0.001) (Figure 3A). The production of violacein pigment by *C. violaceum* (CV12472) was mediated by AHL-mediated QS system. The quantitative assessment of violacein pigment production in biosensor strain C. violaceum CV12472 clearly indicated that the anti-QS activity of AgNPs was dosage dependent. This QS-mediated inhibition of violacein by AgNPs does not affect the bacterial growth, indicating least possibility of bacterial resistance development. Previous studies have also reported the reduced violacein production by silver nanowires (80% reduction) (Pal et al., 2007) and microfabricated AgNPs (100% inhibition) (Singh et al., 2015).

### AgNPs Inhibit Exopolysaccharide (EPS) and Alginate Production

Formation of biofilm plays an important role during the bacterial pathogenesis. Here, we found that AgNPs have anti-QS activity and that known to regulate biofilm formation. Therefore, we further explored the potential of AgNPs as anti-biofilm agents against different bacterial strains by estimating the inhibition of EPS and alginate production. Biofilms are enclosed in a complex EPS network, which facilitates the initial attachment of bacteria to the surface and enhances microbial resistance to antibiotics (Albus et al., 1997). Thus, reduction of EPS production may expose pathogens to antibiotics, thus making them susceptible and hence help in the removal of biofilms. In the present study, we found that EPS extracted from treated and untreated cultures showed concentration dependent decrease, i.e., EPS production was reduced with increasing AgNPs concentration (Figure 3B). The Gram -ve bacteria *P. aeruginosa*, *E. coli*, *C. violaceum*, and *K. pneumoniae* exhibited 12–71, 21–75, 9–61, and 15–79% (*P* < 0.05) inhibition of EPS in the presence of AgNPs respectively, whereas Gram +ve pathogen *S. aureus* showed 31–84% (*P* < 0.05) decrease in EPS production at different sub-MICs (Figure 3B). Our results corroborate well with earlier reports on copper nanoparticles that reduced EPS production by 92% in clinical strains of *P. aeruginosa* (LewisOscar et al., 2015).

Alginate is a major constituent of EPS, and its production protects bacteria from the harsh environment. It helps the attachment of bacteria to the surface and protects them from host immune response and thus making them resistant to antimicrobials. Thus, reduction in alginate production is bound to decrease resistance among the pathogens. Here, we extracted alginate from treated and untreated cultures of bacterial strains and quantified by taking OD at 530 nm. As shown in Figure 3C, AgNPs reduced the alginate production of *P*. *aeruginosa* in a concentration-dependent manner. Synthesized AgNPs showed 26.3–75.6% reduction in alginate production of *P*. *aeruginosa* at concentrations of 1–8 μg/mL (Figure 3C). In an earlier study, significant inhibition of alginate by AgNPs synthesized from metabolites of *Rhizopus arrhizus* has been reported (Cooper and Spitzer, 2015).

### Swarming Mobility Suppressed by AgNPs

Swarming motility plays a key role in the attachment of cells to the surface during biofilm formation and is an important virulence factor of many human and foodborne pathogens. Therefore, impairment of motility will certainly affect biofilm formation adversely. Results of the swarming motility assay have been summarized in Table 2. Concentration dependent decrease in swarming diameter was recorded, and maximum inhibition was recorded at respective 1/2 × MIC against all the test bacterial pathogens. The synthesized AgNPs demonstrated 32–76, 42–81, 18–65, and 40–68% reduction in motility behavior of *C. violaceum*, *P. aeruginosa* PAO1, *E. coli*, and *K. pneumoniae*, respectively at concentrations ranging from 1/16 × MIC- 1/2 × MIC (Table 2). It is also evident from Table 2 that the flagellar driven migration of AgNPs treated test pathogens was impaired considerably as compared to untreated control. Hence, it is envisaged that the biosynthesized AgNPs tend to reduce the biofilm formation in test pathogens by interfering with bacterial ability to reach substratum. Results obtained in the present study are in accordance with the findings on green zinc oxide nanostructures synthesized from *Nigella sativa* seed extract (Al-Shabib et al., 2016). They demonstrated broad-spectrum, statically significant reduction in swarming migration of human and food-borne pathogens *viz. C. violaceum*, *P. aeruginosa* PAO1, *E. coli* and *L. monocytogenes* at tested sub-MICs (Al-Shabib et al., 2016).

**Table 2:** Effect of AgNPs on swarming motility of bacterial pathogens.

Bacterial pathogens	Diameter of swarming migration (mm)				
	
	Control	1/16 × MIC	1/8 × MIC	1/4 × MIC	1/2 × MIC
*P. aeruginosa*	54 ± 3.7	31 ± 2.6	23 ± 2.4	14 ± 1.3	10 ± 0.9
*E. coli*	38 ± 0.9	31 ± 1.7	24 ± 2.1	20 ± 2.5	13 ± 1.8
*C. violaceum*	43 ± 3.2	29 ± 2.1	18 ± 1.7	15 ± 0.6	10 ± 1.6
*K. pneumoniae*	35 ± 1.3	21 ± 1.4	16 ± 1.5	13 ± 1.2	11 ± 1.1


### Anti-biofilm Properties of AgNPs

**FIGURE 4:**
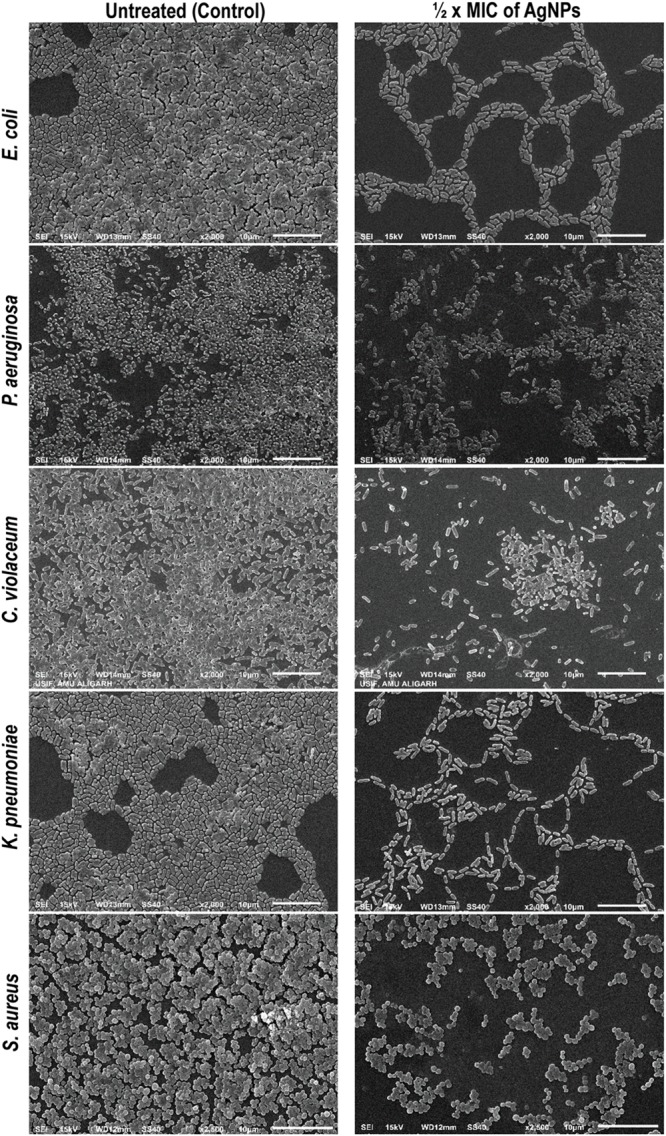
Inhibition of biofilm of *E. coli*, *P. aeruginosa, C. violaceum, K. pneumoniae*, and *S. aureus* by AgNPs under scanning electron microscope (SEM). Biofilm formation by different bacterial agents were assessed by SEM. The biofilm treated by ½ × MIC of AgNPs showed reduction in the biofilm mass production compared with control untreated (scale bar 10 μm).

So far, we have observed that AgNPs inhibited QS-regulated violacein production, alginate, and EPS production, and swarming motility of bacteria. Biofilm formation plays a very crucial role in the pathogenicity of bacteria. In most of the cases, biofilm formation in pathogenic bacteria is regulated by the QS. Thereby, any interference with QS system may regulate the extent of biofilm formation by the pathogen. Furthermore, the biofilm formation was quantified in the presence of AgNPs by measuring absorbance at 470 nm. Data show that AgNPs reduced the biofilm formation in different pathogens at respective sub-MICs (Figure 3D). In the microtiter plate assay, AgNPs exhibited a dose-dependent reduction in the biofilm biomass of pathogens. AgNPs exhibited 22–79, 29–87, 12–59, 22–63, and 17–81% reduction in biofilm biomass of *P*. *aeruginosa*, *E. coli*, *C. violaceum*, *K*. *pneumoniae*, and *S. aureus* respectively at sub-MICs ranging from 1/16 × MIC-1/2 × MIC (Figure 3D). Furthermore, the SEM images also demonstrating the biofilm inhibition by synthesize nanoparticles, grown by different bacterial strains (Figure 4).

Our findings are in the agreement with previous report on polysaccharide-capped AgNPs that demonstrated complete biofilm inhibition in *Bacillus* and *E. coli* at sub-lethal concentrations (Sanyasi et al., 2016). Other studies have also documented biofilm inhibitory properties of AgNPs against bacterial pathogens (Pal et al., 2007).

### AgNPs Attenuate the Toxicity of Liver and Kidney Enzymes *in vivo*

**FIGURE 5:**
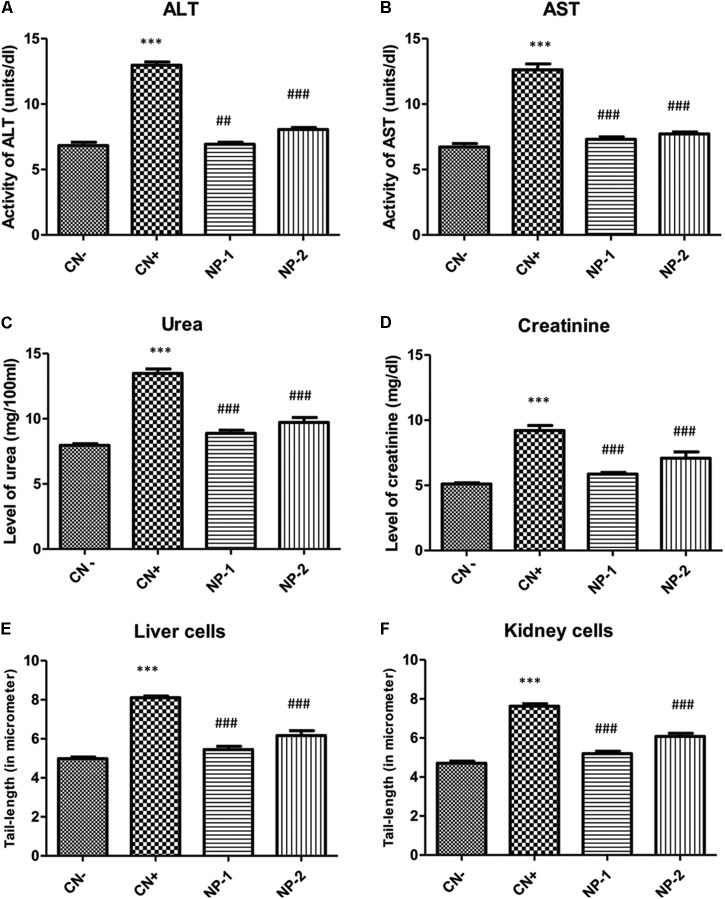
*In vivo* effect of AgNPs on the toxicity of liver and kidney enzymes. Effect of AgNPs on liver and kidney enzymes were assessed to determine, whether synthesized nanoparticle has any cellular toxicity. Data showed **(A)** reduced alanine aminotransferase (ALT) activity, **(B)** aspartate aminotransferase (AST) activity, **(C)** urea level, **(D)** creatinine level in response to the treatment of nanoparticles. **(E)** Comet assay performed to see the effect of synthesized nanoparticles in nuclear-DNA damage. The liver cells treated showed a decrease in the length of DNA damage tail by 32.84 and 23.95% at two different dosage (1 and 2 mg/kg respectively) with respect to positive control. **(F)** Kidney cells, also showed the same effect of the reduction (31.75 and 20.34%) at two different dosage (1 and 2 mg/kg respectively) in olive tail movement indicative of DNA damage (*P* < 0.05, one-way ANOVA with Dunnet post-test in comparison between positive control and nanoparticle treatments). ^∗∗∗^indicate the significant differences in comparison to negative control (CN-), with *p* vale < 0.005. ^###^indicate the significant differences in comparison to positive control (CN+), with *p* vale < 0.005; ^##^indicates significantly different from control positive (CN+) at *p* < 0.05.

To check the suitability of AgNPs as a drug or drug adjuvant, we were interested to know, weather the synthesized nanoparticle has any toxic insult or not. Therefore, we determined the effect of AgNPs on liver and kidney enzymes. Alanine transaminase (ALT) and aspartate transaminase (AST) are chief toxicity markers for the liver. In the present study, ALT activity was elevated by 90.17% in the positive control (CCl_4_- treated animals) with respect to the control, while CCl_4_ used as a positive control (Boll et al., 2001; Ebaid et al., 2014). However, groups- NP-1 and NP-2 demonstrated a decrease in its activity by 46.64 and 37.93% with respect to the positive control (CN+) group (Figure 5A). AST activity was also enhanced in CN+ by 88.07% as compared to the CN- whiles the groups- NP-1 and NP-2 showed a decrease in activity by 42.15 and 38.82% with respect to CN+ group (Figure 5B). The decrease in the activity of both liver function markers indicates that the tolerability of AgNPs. It’s known, if the liver function markers are slightly higher after the treatment of synthesized compound or drug, then such compound or drug is considered as tolerable.

Similarly, urea and creatinine are considered as the key kidney function markers for assessment of the toxicity of any compound or drug *in vivo*. In the present study, the level of urea was raised by 69.56% in CN+ group as compared to the negative control (CN-) group. However, groups- NP-1 and NP-2 showed a decline in its level by 34.19 and 27.96% respectively in comparison to CN+ group (Figure 5C). Similarly, the creatinine level was also enhanced in CN+ by 80.58% with respect to the CN- group. Hitherto, groups- NP-1 and 2 showed low level as of 36.26 and 23.23%, compared to the CN+ group (Figure 5D). It is widely accepted that any drug/chemical after undergoing metabolized in the body are excreted out by the kidneys with the urine. If the agent is toxic, then the renal function markers (urea and creatinine) are elevated that is functional to its toxicity. The decrease in both the studied markers post-treatment with the NPs as compared CN+ indicates that these particles exert a very low degree of toxicity in the kidneys.

Sharma et al. (2012) have documented that NPs generate reactive oxygen species *in vivo* that can further damage the target organs and various cellular components including mitochondria. In the present study, none of the treated animals was dead after completion of the treatment at the taken doses. Besides, there was not any significant decrease in body weight or physical appearance (data not shown) in the NPs-treated animals. Furthermore, the liver and renal function tests also showed mild toxicity with increasing the dose of NPs as compared to CN- group. Also, the decrease in both the organ function markers with the CN+ group indicates that the NPs are quite tolerable in the rats. It is believed that if any chemical/compound doesn’t affect the function of liver and kidney, then such compound is suitable as a drug-carrier or as an adjuvant (Hassan et al., 2010). Besides, improvement in liver and renal markers in the NPs-treated groups as compared to CN+ entails healing property of the particles.

### AgNPs Inhibit DNA Damage

**FIGURE 6:**
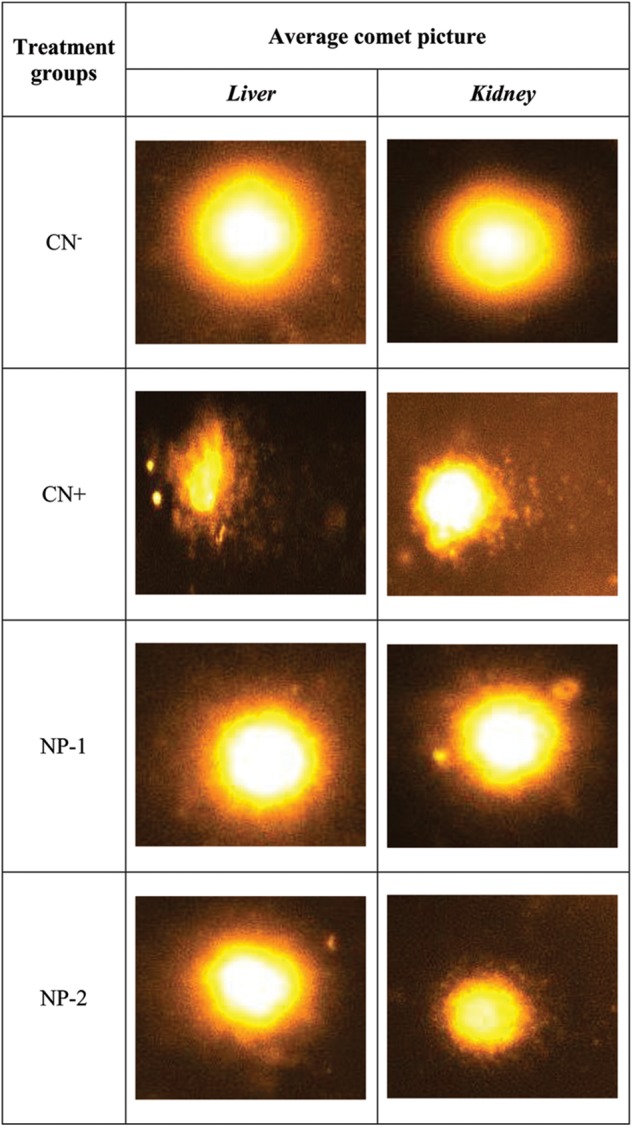
Synthesized AgNPs inhibit DNA damage. Representative images of the comet assay to estimate the DNA damage in cells treated with nanoparticles. Different groups were assigned as negative control (CN–), positive control (CN+), nanoparticle treated; dosage 1 mg/kg (NP-1), and nanoparticle treated; dosage 2 mg/kg (NP-2) in liver and kidney cells.

We further evaluated the effect of AgNPs-induced nuclear DNA damage by comet assay (Figure 5E,F, 6). In the liver cells, the olive tail length of CN+ group was found to be increased by 62.97% as compared to the CN- group. However, groups- NP-1 and NP-2 showed a decrease in the length by 32.84 and 23.95% with respect to CN+ (Figure 5E, 6). Similarly, in kidney cells, CN+ group showed an increase in its olive tail length by 62.13% with respect to the control. Hitherto, groups- NP-1 and NP-2 exhibited a decline in the length by 31.75 and 20.34% respectively as compared to the CN+ (Figure 5F, 6). Comet assay results supported the biochemical toxicity profiling of the nanoparticles. It is also noteworthy that the nanoparticles were more of the hepatotoxicant than the nephrotoxic. Also, the toxicity of the nanoparticles was not dose-dependent that entails the NPs are suitable to be used as an adjuvant even at the higher doses with the established or the new drugs. These results are harmonious with the liver and renal function tests in which also the NPs demonstrated mild toxicity under the tolerable range in both the target organs.

Previous studies reported that AgNPs have excellent antibacterial, antiviral and anti-inflammatory properties. Furthermore, recently their anticancer activity is among the hot springs of contemporary research (Zhang et al., 2016; Rónavári et al., 2017). The present preliminary toxicity profiling of these particles are aimed at exploring their therapeutic window against various challenges in healthcare and environmental problems. In the present work, it is crystal clear that the nanoparticles are mildly toxic *in vivo* those were found tolerable at the moderate dose in the living system.

### Homology Modeling and Structure Assessment of Enzymes Involved in QS

The molecular docking studies were employed to gain an insight into the mechanism involved in the inhibitory action of Ag on the crucial enzymes of QS. The information about the three-dimensional structure of a protein is essential to visualize its biological function in the native environment (Rehman et al., 2015). In the present study, we have modeled the three-dimensional structures of RhlR and PqsA using I-TASSER (Roy et al., 2010) as the query sequences have similarity index of less than 25% with the target sequence. The generated models were verified for their accuracy by Ramachandran plot using RAMPAGE and also by SAVES and QMEAN tools (Pontius et al., 1996; Lovell et al., 2003). The three-dimensional structures of RhlR and PqsA after modeling with I-TASSER have been shown in Supplementary Figure S5. I-TASSER had predicted that the C-score of proteins RhlR and PqsA were 1.23 and 1.32, respectively (Supplementary Table S1). For a good prediction, the C-score usually varies between +2 and -5 wherein the higher value of C-score represents a good quality of the structure prediction. Similarly, the estimated RMSD and TM-score of RhlR and PqsA were 4.8 ± 3.1 and 0.88 ± 0.07 and 3.1 ± 2.2 and 0.90 ± 0.06, respectively (Supplementary Table S1). The Ramachandran plot of RhlR showed that 87.4% of the residues occupied the favored region and 10.1% residues occupied allowed region while only 2.5% of the residues were in the outlier region (Supplementary Figure S6A). Similarly, the Ramachandran plot of PqsA showed that the residues in the favorable, allowed and outlier regions were 84.7, 11.7, and 3.6%, respectively (Supplementary Figure S6B). Together these results show that the modeled structures of RhlR and PqsA produced by I-TASSER had a good three-dimensional structure. The quality of the modeled structures was also accessed by ERRAT plots wherein Supplementary Figures S7A,B represent the ERRAT plots of RhlR and PqsA, respectively. A good resolution structure has overall quality factor around 95% or higher. Supplementary Figure S8 shows that 95.279% of the residues of RhlR and 94.466% of the residues of PqsA fall below 95% rejection limit thereby confirming that they have a three-dimensional structure whose resolution matches the experimentally determined structures (resolution below 2.5 Å) by X-ray crystallography. Moreover, we also determined the ability of the primary amino acid sequence to attain a correct three-dimensional structure by analyzing Verify 3D plots (Supplementary Figure S8). Our results indicate that 86.31 and 97.49% of the residues of RhlR and PqsA have successfully attained proper three-dimensional structure. The quality of the modeled structures of RhlR and PqsA were also accessed by QMEAN tools, and the results are presented in Supplementary Table S2. We found that the Z-score of a total QMEAN parameter of RhlR and PqsA were -1.79 and -0.70 signifying a good quality structure (Supplementary Table S2).

### Molecular Docking Determine the AgNP’s Binidng Capacity to Various Enzymes

**FIGURE 7:**
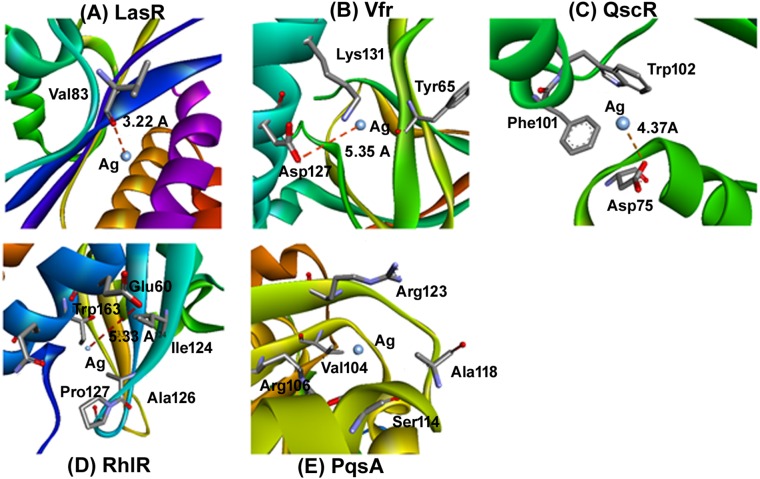
Molecular docking determine the AgNP’s binidng capacity to various enzymes. Molecular docking was performed to idnetify the interaction of synthesized nanoparticles with amino acid residues. The docking model show an interaction of Ag to LasR through Val83 and Vfr through Tyr65, Asp127, and Lys131. further RhlR interacted with Ag through Ile124, Ala126, Pro127, Glu160, Thr163, and Gln164. Similarly the docking of Ag with **(A)** LasR, **(B)** Vfr, **(C)** QscR, **(D)** RhlR, and **(E)** PqsA.

In the next step, to assess and predict the biological interaction of nanoparticles, we performed the molecular docking using Patch Dock server for the three-dimensional structures of LasR, Vfr, QscR, RhlR, and PqsA for (Hassan et al., 2012). The amino acid residues that interact with Ag and the type of interactions are presented in Figure 7. It is clear that Ag interacted to LasR through Val83 while it was bound to Vfr through Tyr65, Asp127, and Lys131 (Figure 7). An electrostatic interaction (5.35 Å) between Ag and Asp127 of Vfr played a significant role in stabilizing the complex. Moreover, Asp75, Phe101, and Trp102 of QscR were found to interact favorably with Ag. The QscR-Ag complex was stabilized by an electrostatic interaction between Ag and Asp75 (Figure 7). Similarly, RhlR interacted with Ag through Ile124, Ala126, Pro127, Glu160, Thr163, and Gln164. The RhlR-Ag complex was stabilized by an electrostatic interaction between Ag and Glu160 (5.33 Å). On the other hand, hydrophobic interactions were crucial for stabilizing complex between Ag and PqsA. The amino acid residues that participate in the hydrophobic interactions were Val104, Arg106, Ser114, Ala118, and Arg123 (Figure 7). We infer from the molecular modeling and docking studies that Ag can effectively bind to various enzymes that are crucial for mediating QS-controlled bacterial pathogenesis.

## Conclusion

**FIGURE 8:**
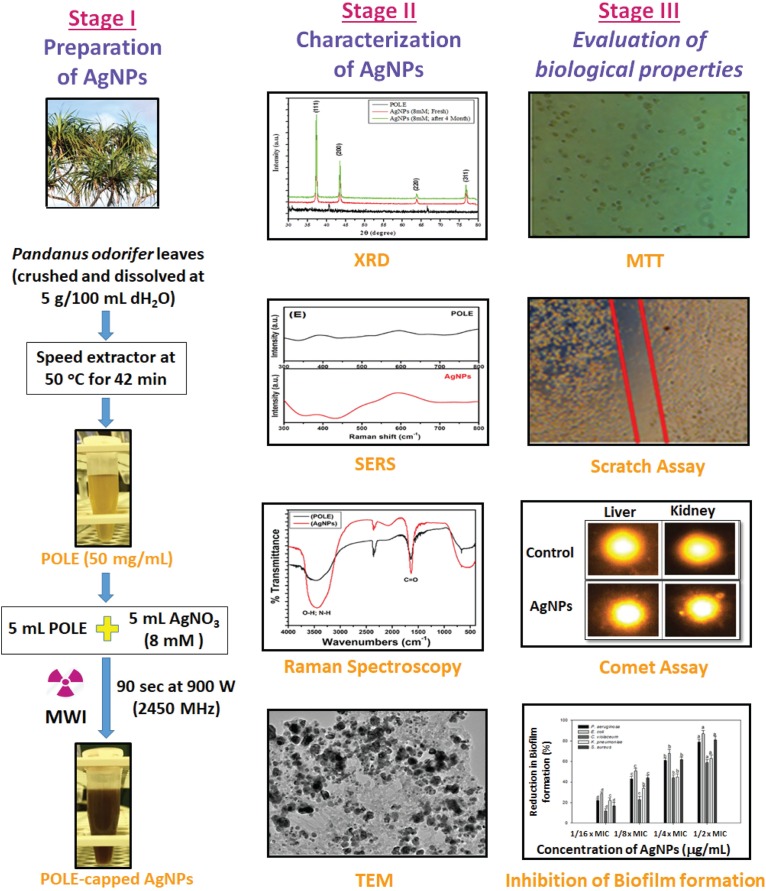
Model showing the systematic approch for the synthesis of nanoparticle (AgNPs) and their characterization. Three stages of the model showing the basic steps for the synthesis of silver nanoparticles (AgNPs), their characterization to confirm the nature of nanoparticles and biologcal functions. As the AgNPs have anti-biofilm and anti-cancer propertise, the synthesized AgNPs could be further tested for many therapeutics options and clinical managements.

The POLE-capped silver nanoparticles have been successfully synthesized from AgNO_3_ and *P. odorifer* leaf extract using microwave irradiation method and were evaluated for anti-metastasis and anti-biofilm potentials. The overall synthesis procedure and different aspect to characterize the AgNPs and their functionality are shown in study model Figure 8. These AgNPs were highly crystalline in nature with FCC structure of 5–9 nm size. MTT assay of synthesized AgNPs displayed significant anti-cancer activity for RBL cancer cells. Moreover, scratch assay re-affirmed their anti-metastasis potential by inhibiting the migration of cancer cells. The POLE-capped AgNPs exerted low degree of toxicity *in vivo* and were found well-tolerable at a moderate dose. Hence, they are suitable to be used in the form of adjuvants or drug–carrier as highly promising anti-microbial and anti-cancer candidates. Moreover, these NPs showed excellent anti-bacterial properties against various Gram +ve and Gram -ve bacterial strains. The prepared AgNPs showed anti-QS activity by inhibiting violacein and alginate productions by 89.6 and 75.6% respectively. Further the EPS production and swarming mobility were significantly decreased in the presence of AgNPs. These are the key factors involved in the initial attachment and maturation of biofilm. *In silico* molecular modeling and docking studies further confirmed the inhibition of key enzymes involved in QS. The POLE-capped AgNPs synthesized in this study can be further evaluated for their anti-cancer and anti-bacterial activities and optimized for industrial scale production. Further studies are also warranted to determine the efficacy and dose response of POLE-capped AgNPs for clinical applications. Moreover, these AgNPs can be tapped for the development and fabrication against colonization of drug-resistant pathogenic micro-organisms on medical devices such as catheters, tubes, probes, dressings, etc.

## Author Contributions

AH and MA conceived and designed the experiments. AH, SP, MAK, MR, IH, FH, MSK, RK, FA, and GS performed the experiments and analyzed the data. MA, AH, MR, SA, MAK, and SP wrote the manuscript. All authors reviewed and approved the final version of the manuscript.

## Conflict of Interest Statement

The authors declare that the research was conducted in the absence of any commercial or financial relationships that could be construed as a potential conflict of interest.

## Footnotes

1 https://www.ncbi.nlm.nih.gov/

2 http://www.rcsb.org/pdb/home/home.do

3 https://pubchem.ncbi.nlm.nih.gov/
