# Supplementary material for: Biosynthesized Silver Nanoparticle (AgNP) From Pandanus odorifer Leaf Extract Exhibits Anti-metastasis and Anti-biofilm Potentials
Source: Front Microbiol. 2019 Feb 12;10:8. doi: 10.3389/fmicb.2019.00008 (PMC6396724; doi:10.3389/fmicb.2019.00008)
Supplement: Supplementary file 1 [file Data_Sheet_1.docx]

**Supplementary information**

**Biosynthesized Silver Nanoparticle (AgNP) from *Pandanus odorifer* exhibits Anti-Metastasis and Anti-Biofilm Potentials**

Afzal Hussain^1^, Mohamed F. Alajmi^1^, Meraj A. Khan^2#^, Syed A. Pervez^3^, Faheem Ahmed^4^, Samira Amir^4^, Fohad M. Husain^5^, Mohd S. Khan^6^, Gouse M. Shaik^6^, Iftekhar Hassan^7^, Rais A. Khan^8^, Md. Tabish Rehman^1#^

^1^Department of Pharmacognosy, College of Pharmacy, King Saud University, Riyadh-11451, Kingdom of Saudi Arabia.

^2^Program in Translational Medicine, Peter Gilgan Centre for Research and Learning, The Hospital for Sick Children, Toronto, ON, Canada.

^3^Helmholtz Institute Ulm, Electrochemical Energy Storage, Helmholtzsteabe, 11, 89081, Ulm, Germany.

^4^Department of Chemistry, College of Science & General Studies, Al Faisal University, Riyadh, Kingdom of Saudi Arabia.

^5^Department of Food Science and Nutrition, College of Food and Agriculture, King Saud University, Riyadh-11451, Kingdom of Saudi Arabia.

^6^Protein Research Chair, Department of Biochemistry, College of Science, King Saud University, Riyadh 11451, Kingdom of Saudi Arabia.

^7^Department of Zoology, College of Science, King Saud University, Riyadh 11451, Kingdom of Saudi Arabia.

^8^Department of Chemistry, College of Science, King Saud University, Riyadh 11451, Kingdom of Saudi Arabia.

**Running title:** Anti-cancer and anti-biofilm properties of biosynthesized silver nanoparticles.

**Key words:** Silver nanoparticles (AgNPs), anti-metastasis, anti-biofilm, quorum sensing, molecular docking

**^#^Corresponding authors:**

**Dr. Meraj Alam Khan**

Program in Translational Medicine

The Hospital for Sick Children Research Institute

Peter Gilgan Centre for Research and Learning

686 Bay Street, Toronto, ON, M5G 0A4

Tel: 416-813-7654 ext.303388

Fax: 416-813-5771

Email: meraj.khan@sickkids.ca

**Dr. Md Tabish Rehman**

Department of Pharmacognosy,

College of Pharmacy,

King Saud University,

Riyadh 11451, Kingdom of Saudi Arabia

Tel: +966556814200, Fax: +96614677245

E-mail: [mrehman@ksu.edu.sa](mailto:mrehman@ksu.edu.sa),

**Table S1. Quality assessment parameters of the predicted RhlR and PqsA models**

| **Name of the Protein** | **C-Score** | **Estimated TM-Score** | **Estimated RMSD (**Å**)** |
| --- | --- | --- | --- |
| **RhlR** | 1.23 | 0.88 ± 0.07 | 4.8 ± 3.1 |
| **PqsA** | 1.32 | 0.90 ± 0.06 | 3.1 ± 2.2 |

**Table S2. QMEAN assessment parameters for the modeled proteins**

| **Parameters** | **Rhl R** | **Z-Score (RhlR)** | **PqsA** | **Z-Score (PqsA)** |
| --- | --- | --- | --- | --- |
| C-beta interaction energy | -80.62 | -0.59 | -116.17 | -0.96 |
| All-atom pairwise energy | -5745.15 | -0.39 | -10496.80 | -0.93 |
| Solvation energy | -30.28 | 0.62 | -41.49 | -0.38 |
| Torsion angle energy | -22.69 | -2.71 | -82.87 | -2.04 |
| Secondary structure agreement | 83.4% | 0.18 | 81.2% | 0.39 |
| Solvent accessibility agreement | 75.5% | -0.80 | 81.0% | -0.04 |
| Total QMEAN-score | 0.603 | -1.79 | 0.708 | -0.70 |





**Fig. S1. UV-Vis absorption spectra of biogenic AgNPs after four months.**


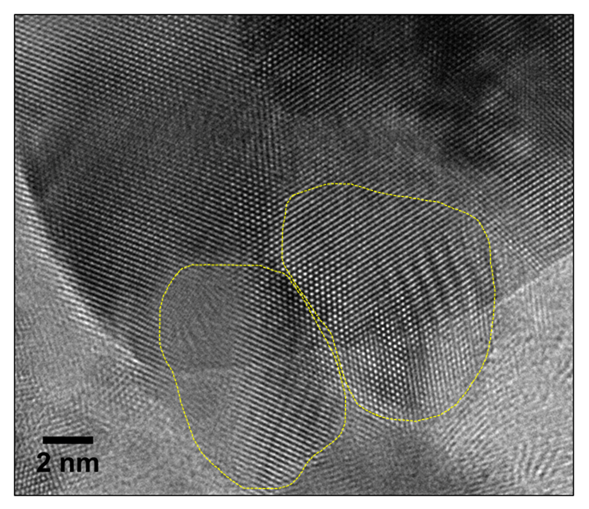


**Fig. S2. HRTEM images of biogenic AgNPs after four months.**


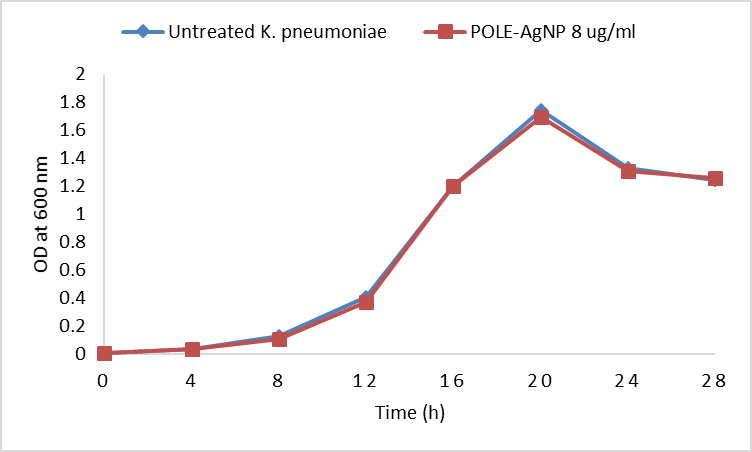

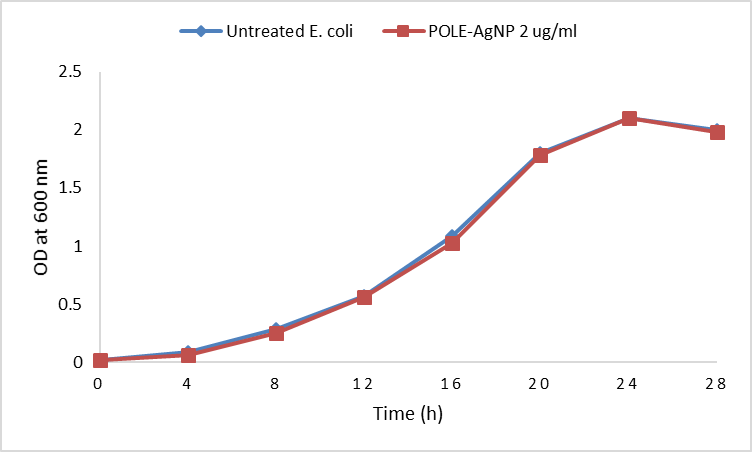

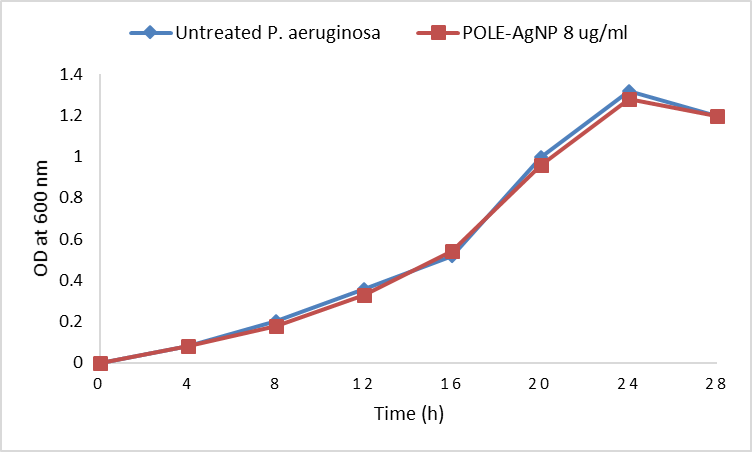

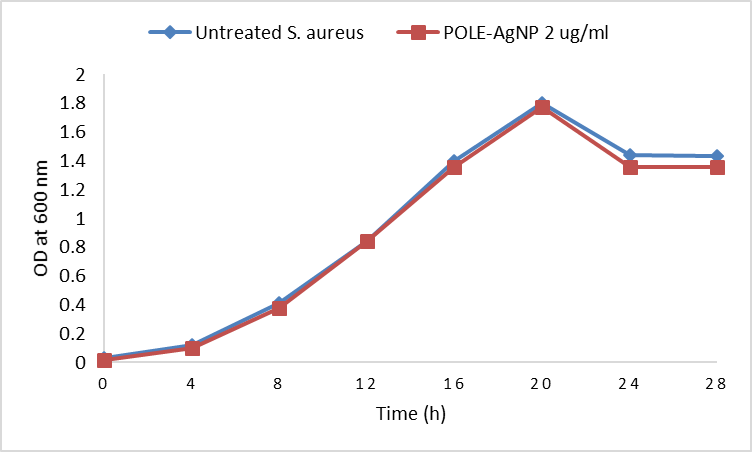

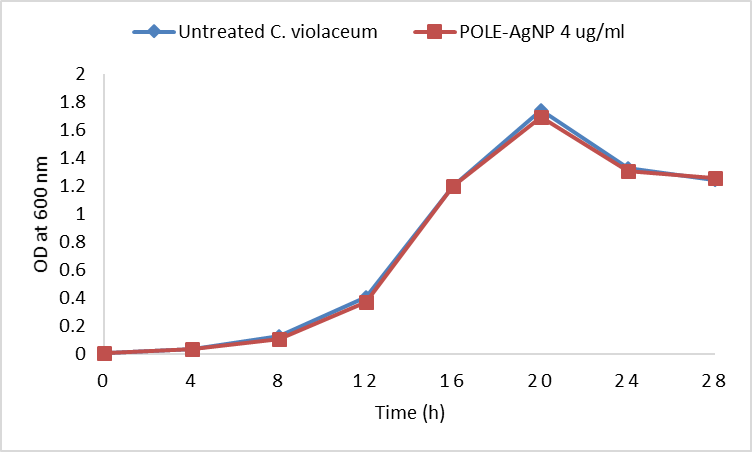


**Fig. S3. Growth curve analysis of test pathogens at respective ½ x MIC.**


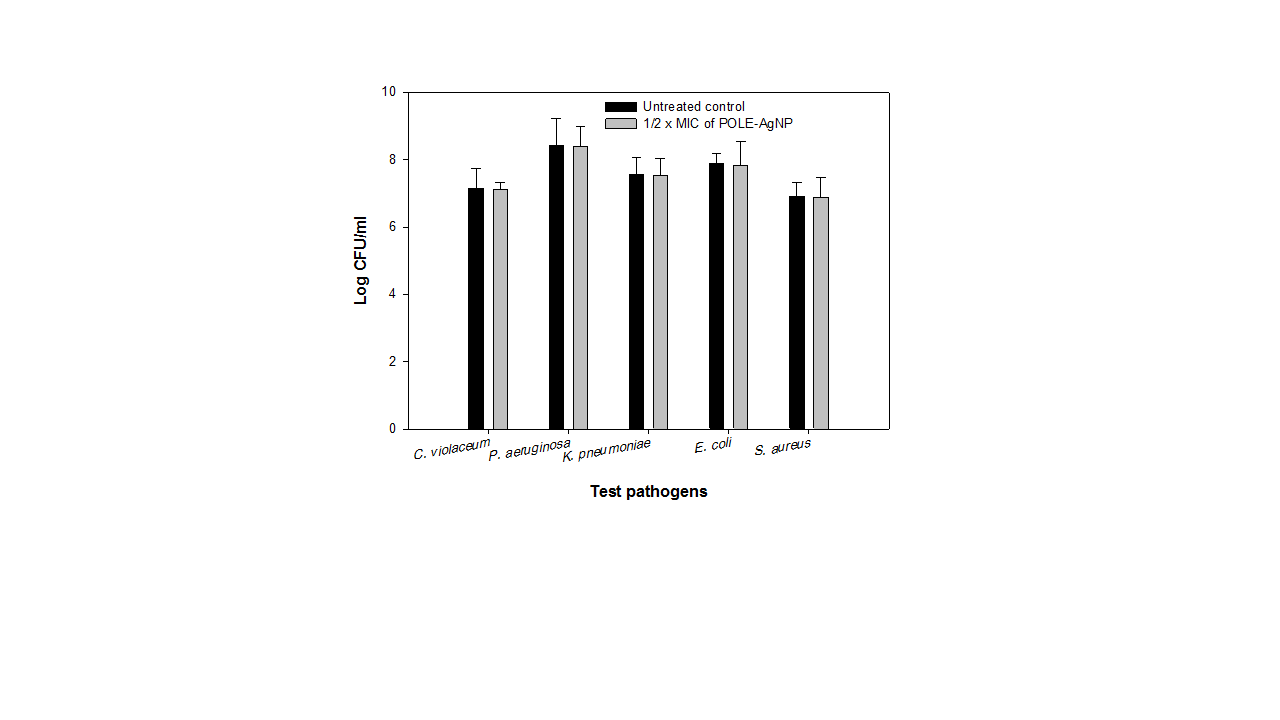


**Fig. S4. Log CFU values of test pathogens at ½ x MICs.**


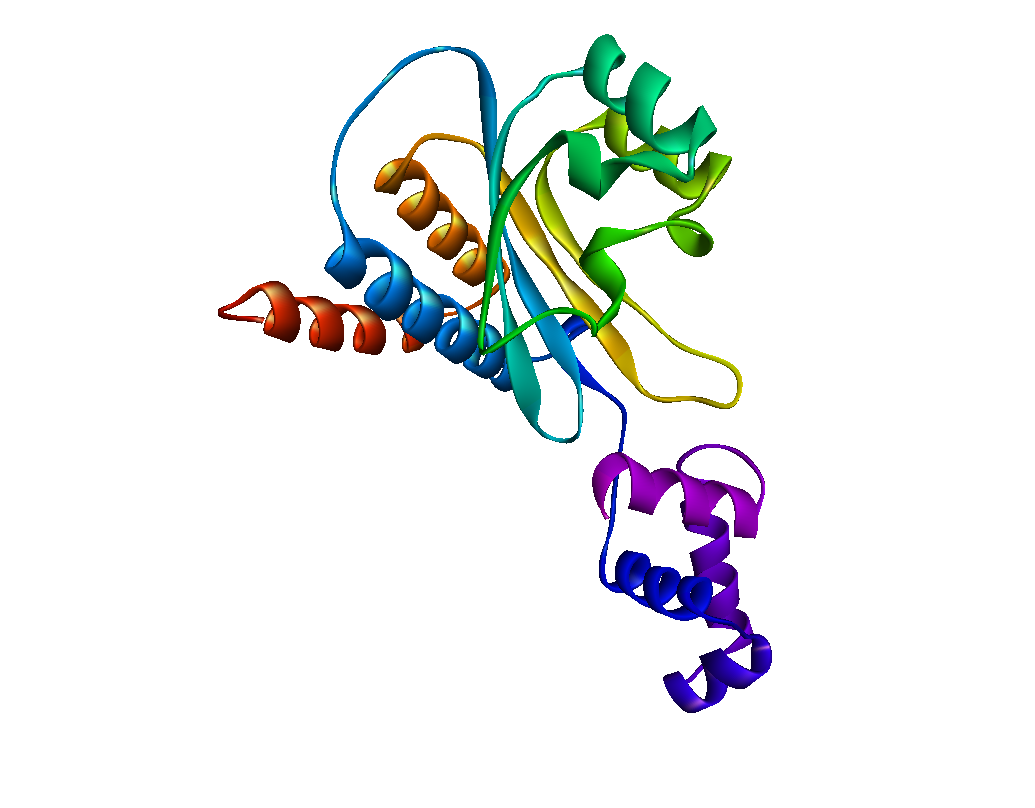

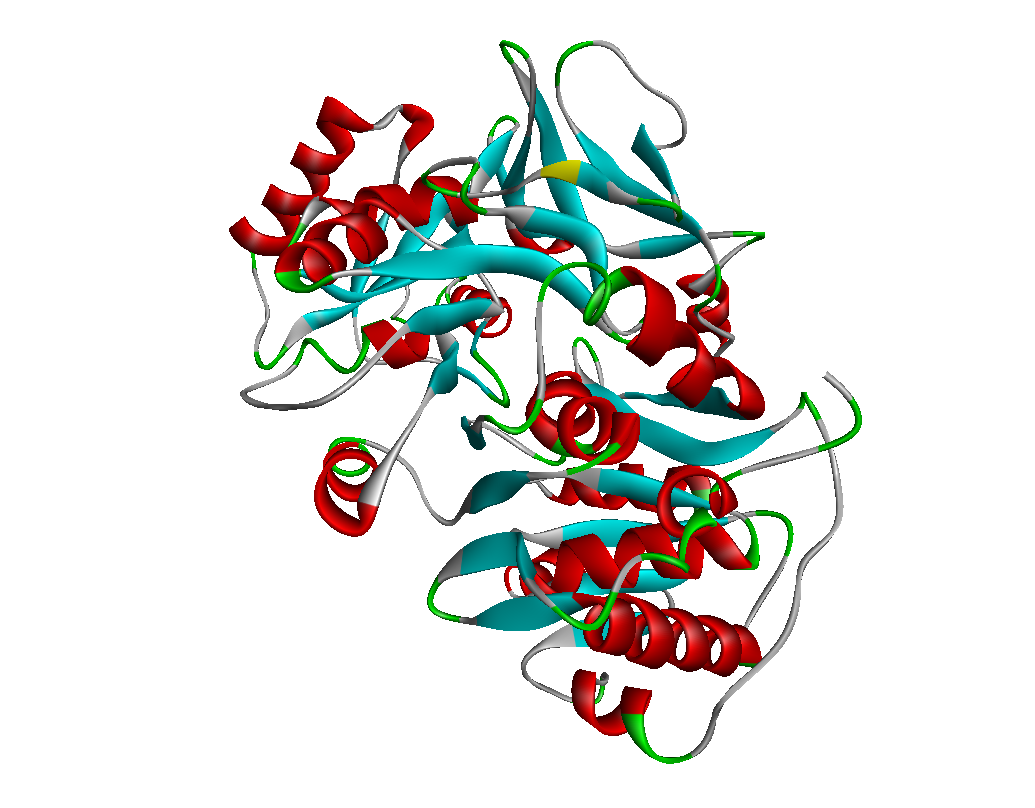


**(A) (B)**

**Fig. S5.** **Modeled three-dimensional structures of (A) RhlR and (B) PqsA**


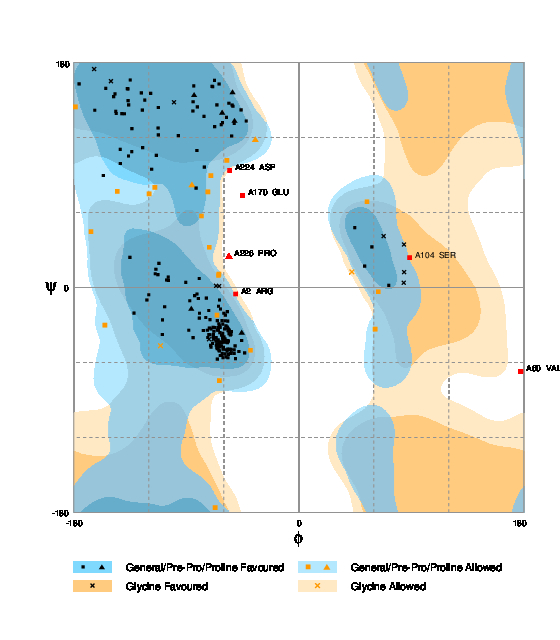

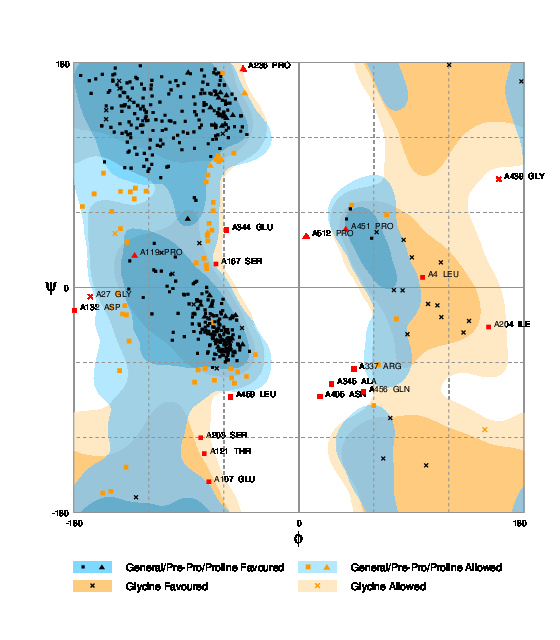


1. **(B)**

**Fig. S6. Ramachandran Plot of (A) RhlR and (B) PqsA**


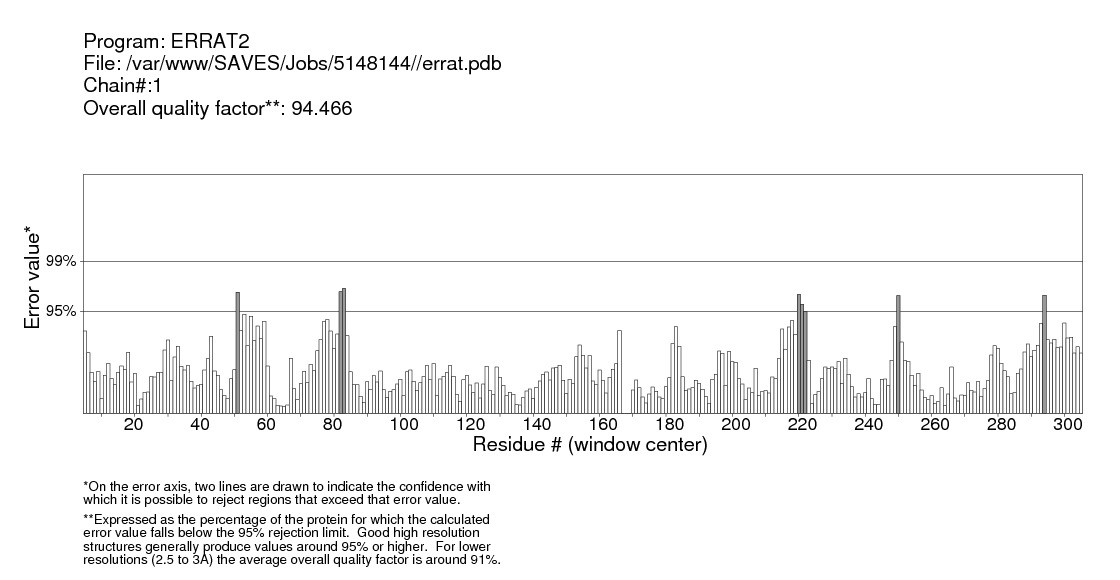

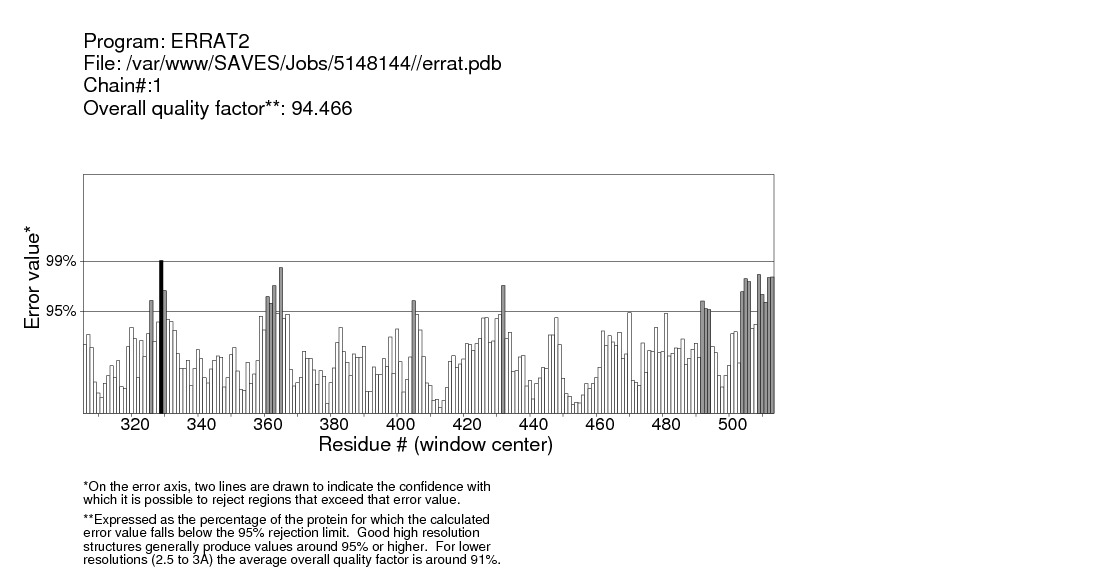

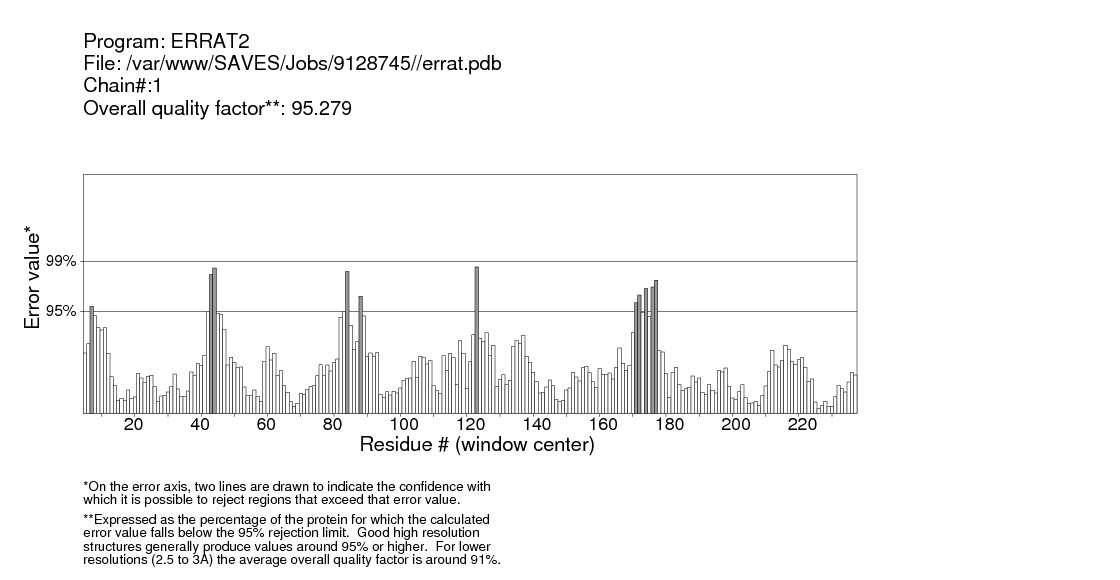


**(A)**

**(B)**

**Fig. S7. ERRAT plots of (A) RhlR and (B) PqsA**


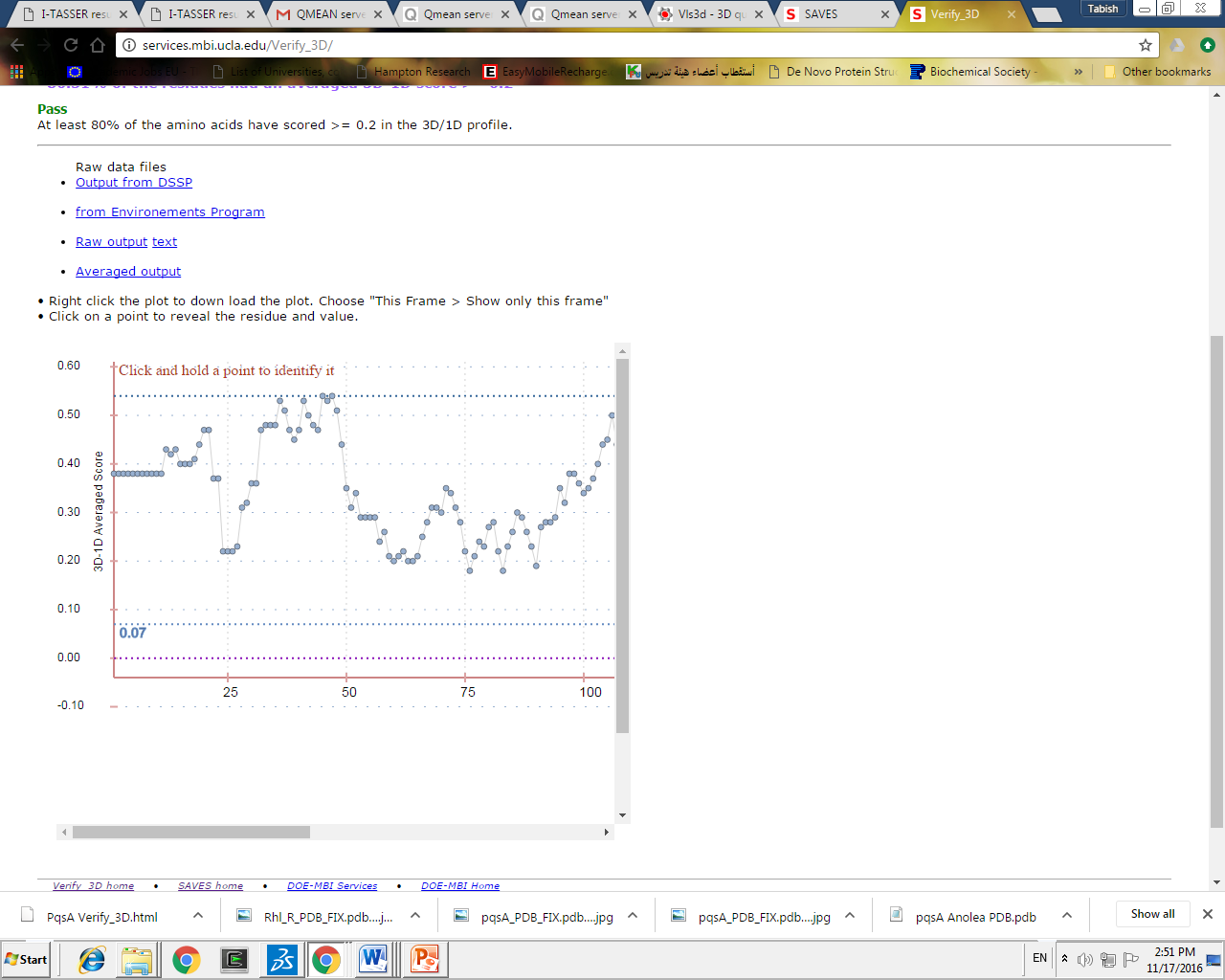

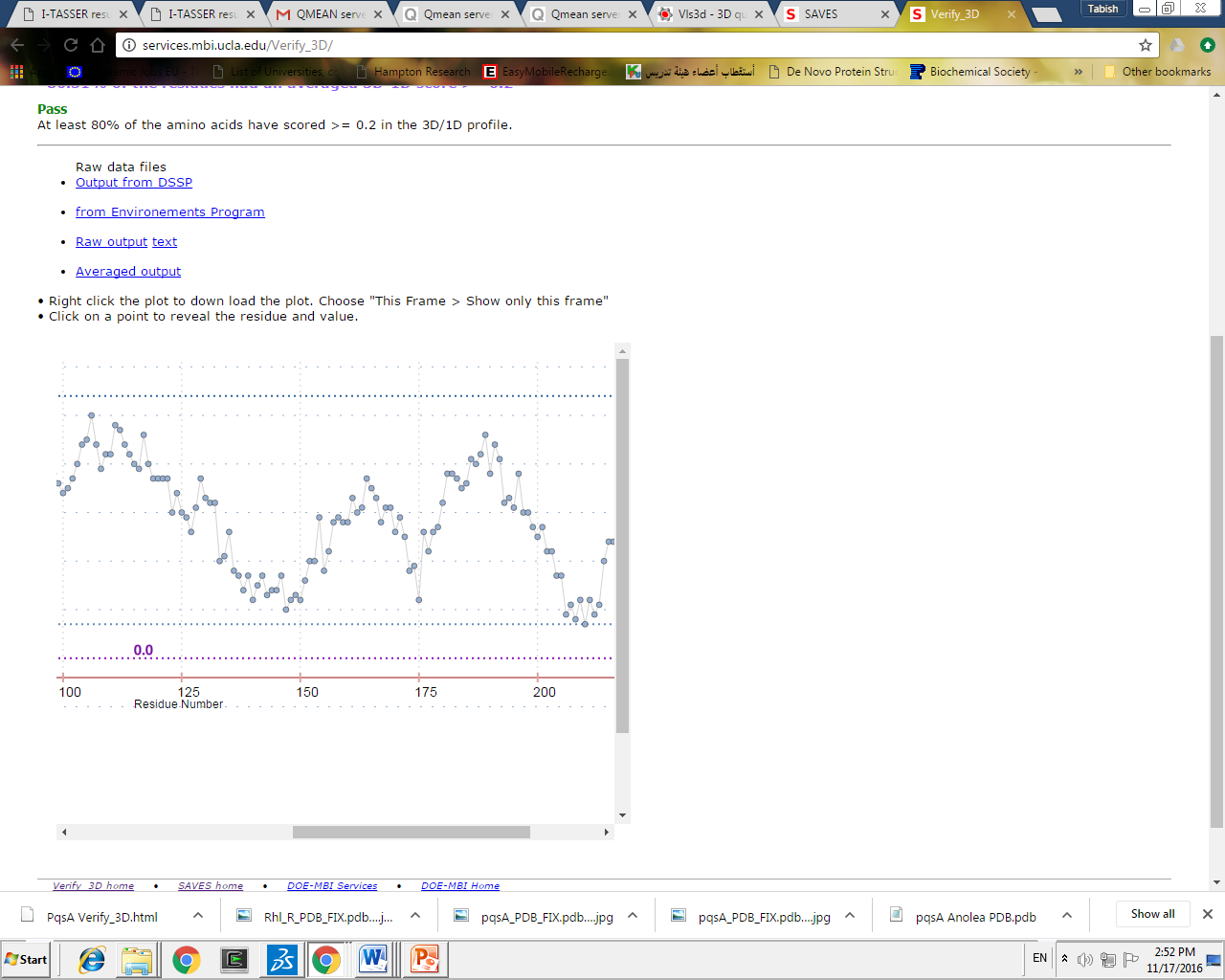

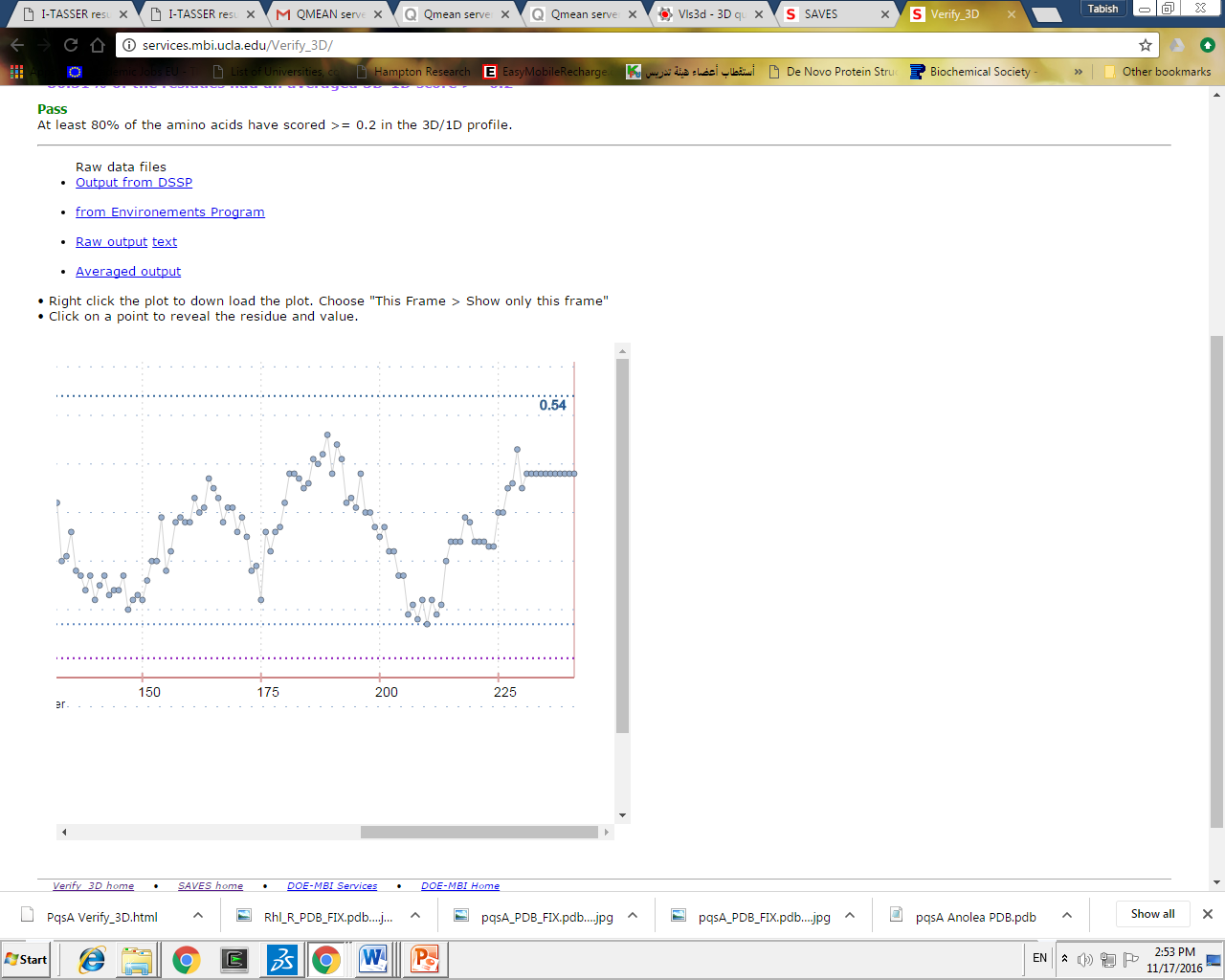


**(A)**


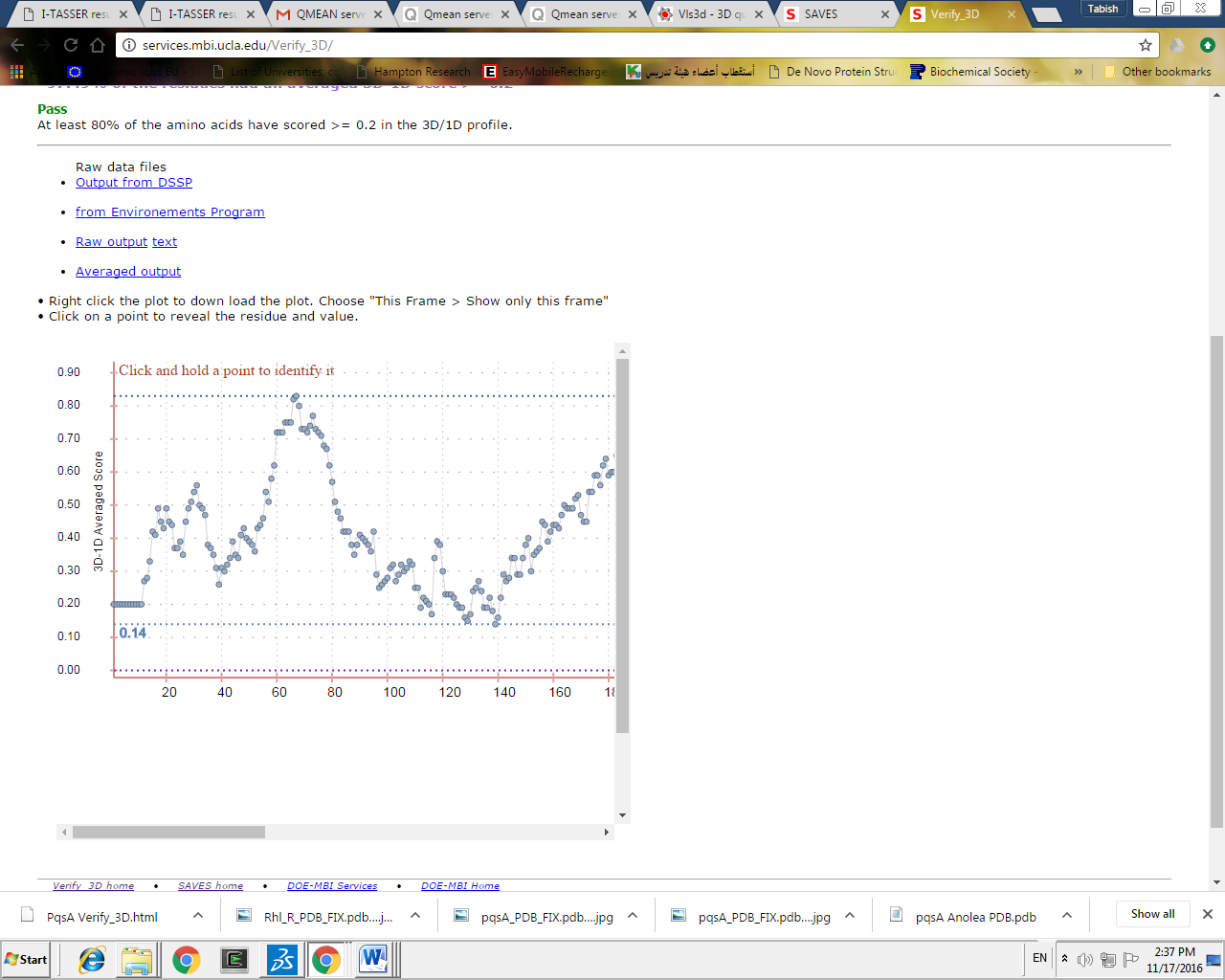

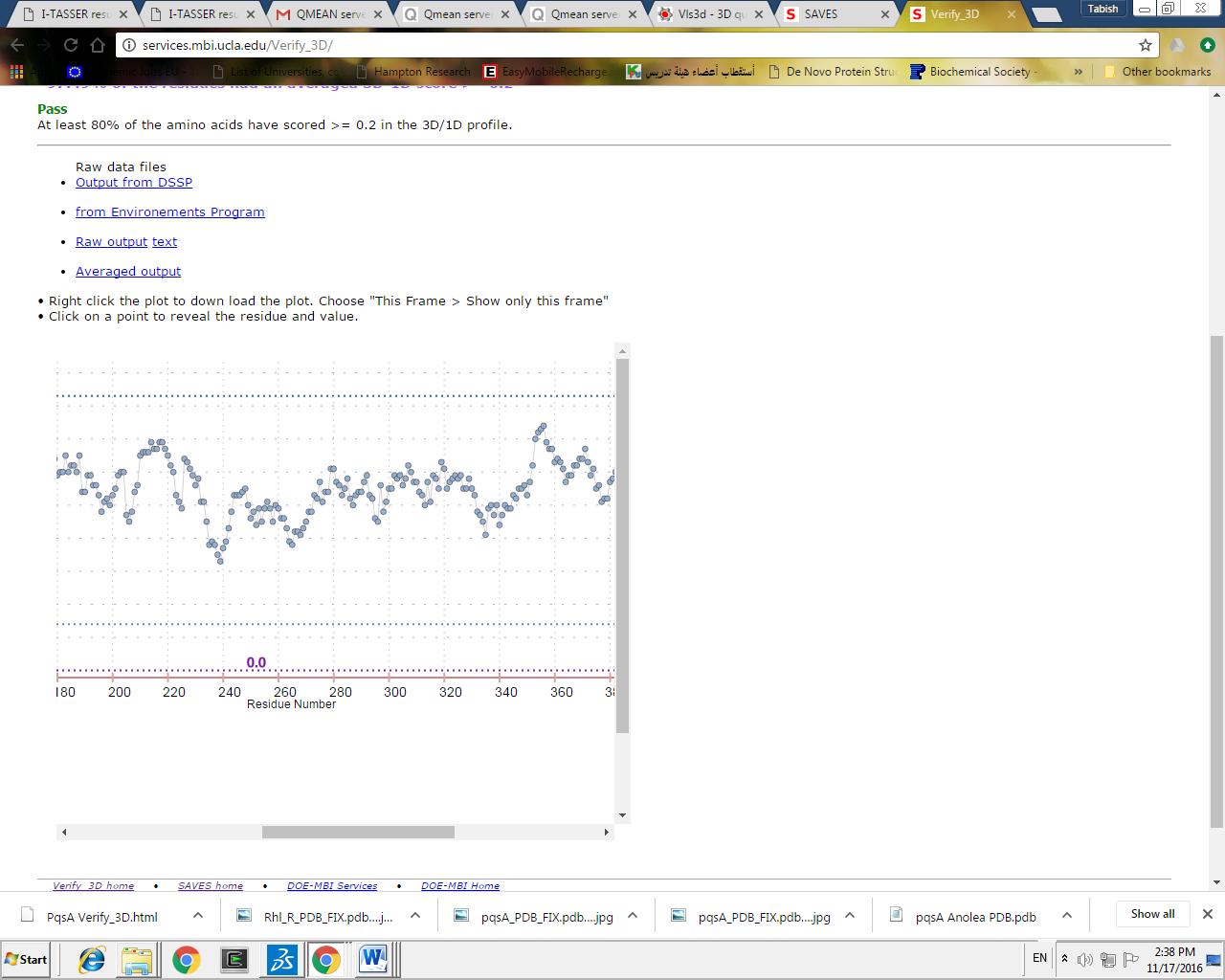

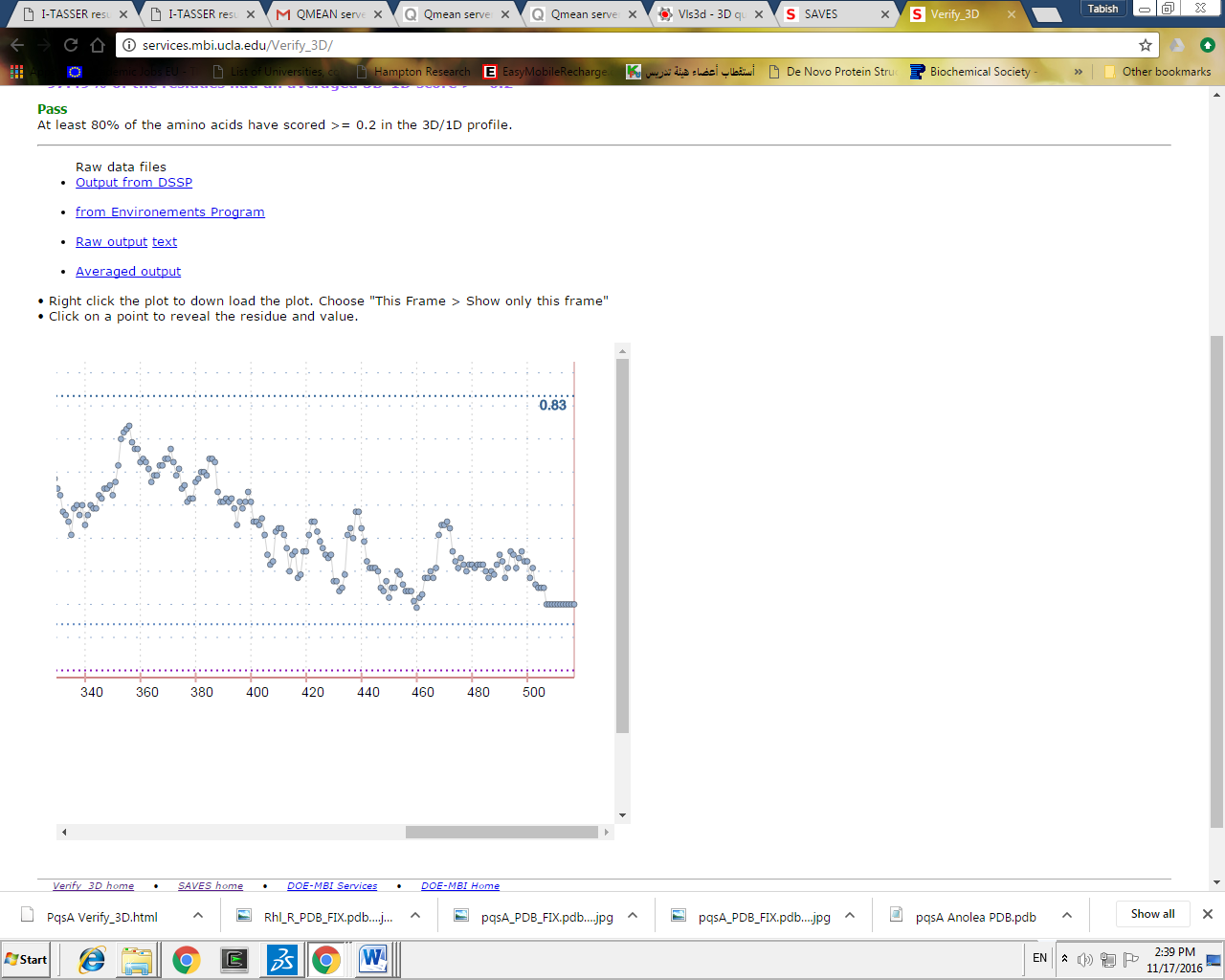


**(B)**

**Fig. S8. Verify 3D plots of (A) RhlR and (B) PqsA**
